# Interpretation of southern hemisphere humpback whale diet via stable isotopes; implications of tissue-specific analysis

**DOI:** 10.1371/journal.pone.0283330

**Published:** 2023-04-03

**Authors:** June Eggebo, Jasmin Groß, Susan Bengtson Nash

**Affiliations:** Southern Ocean Persistent Organic Pollutants Program, Centre for Planetary Health and Food Security, School of Environment and Science, Griffith University, Nathan, QLD, Australia; University of Maryland Center for Environmental Science, UNITED STATES

## Abstract

Blubber and skin are commonly used tissues in stable isotope analysis for the purpose of investigating cetacean diet. Critical comparison of tissue-specific isotopic signals is, however, lacking resulting in uncertainty surrounding the representativeness and therefore utility of different tissues for accurate determination of recent foraging. This study used remotely biopsied blubber and skin tissues from southern hemisphere humpback whales for strategic comparison of δ^**13**^C and δ^**15**^N values. Samples were collected between 2008–2018 as part of long-term monitoring under the Humpback Whale Sentinel Program. Blubber tissues were lipid-extracted prior to analysis, whilst mathematical lipid-correction was performed on skin samples. Isotopic values from paired blubber and skin samples from the same individuals were compared to assess whether tissues could be used interchangeably for isotope analysis and dietary interpretation. Significant differences were observed for both δ^13^C and δ^15^N, flagging previously undocumented methodological considerations, and the need for method validation and standardisation in application of these approaches. This study therefore advances methodological aspects of cetacean dietary analysis. This is of elevated importance in the context of rapidly changing ocean ecosystems.

## 1. Introduction

Southern hemisphere humpback whales (*Megaptera novaeangliae*; SHHWs) have been implemented as a sentinel species for the circumpolar surveillance of pollution and climate change in the Southern Ocean [1, 2]. As capital breeders, these populations rely on intensive summer feeding on Antarctic krill (*Euphausia superba;* hereafter ‘krill’; [3, 4], to sustain their annual winter migrations to lower latitudes for breeding and calving. The narrow foraging niche of SHHWs results in a distilled connection between ecosystem productivity and energetic provisioning (both prey type and foraging success; Castrillon and Bengtson Nash [5]. Their ecophysiology thus renders these populations powerful indicators of ecosystem productivity and change.

Krill are a sympagic species, where sea-ice provides feeding habitats and refuge for early life stages [6, 7]. Polar ecosystems are undergoing rapid change, manifesting in sea-ice melt [8], ocean acidification [9], and a rise in sea water temperature [10]. Kill rely on a stable physio-chemical sea-ice environment and changes can impact krill recruitment and survival [11, 12]. Any change in the abundance and/or availability of krill is expected to carry significant implications for krill consumers [13, 14]. Humpback whale (HW) populations globally show a high degree of plasticity in both their target prey and foraging behaviour [15, 16]. As such, SHHWs may be expected to respond to a change in krill availability through diversified foraging, including changes to both prey and foraging range. Longitudinal monitoring of SHHW diet has therefore been identified as a core sentinel parameter under the Humpback Whale Sentinel Program (HWSP), with interannual variation and drift assumed to reflect a change in krill availability.

Ecologists use bulk stable isotope analysis (BSIA) to directly identify and trace elemental cycling in the biosphere [17]. Over the last few decades, BSIA has played a significant role in research involving animal migration [18, 19], diet [20, 21], reproduction [22, 23] and food web connectivity [24, 25]. The interpretation of bulk stable isotope (BSI) signals is, however, not without uncertainty. In addition to the prey type and foraging range, the trophic position (TP; classification of organisms based on theoretical feeding relationships within an ecosystem) is known to be influenced by endogenous factors such as nutritional stress, metabolic activity of tissues, diet quality, body size, excretory mechanisms and feeding rate [26–30]. Further, the isotopic signals associated with tissues of different biomolecular composition (i.e. lipids, carbohydrates, proteins) have frequently been recorded [30–32].

Stable isotopes of carbon (^13^C,^12^C) and nitrogen (^15^N,^14^N) have, in particular, become valuable in diet research of marine mammals [33–36]. The use of stable isotope analysis to investigate the structure of food webs is based on two assumptions: namely that the isotopic composition of consumer tissue reflects the isotopic composition of their diet, and that consumers are slightly enriched in ^15^N and to a lesser extent in ^13^C compared to their food [37, 38]. The phenomenon is called ‘trophic discrimination’, also referred to as ‘trophic fractionation’ and averages 0.5–1.0 ‰ for carbon (Δ^13^C; [31], and 2–4 ‰ for nitrogen (Δ^15^N; [17, 37, 39]). Trophic levels (TLs) are a hierarchical way of classifying organisms according to their theoretical feeding relationships within an ecosystem [40]. Nitrogen isotopes (δ^15^N) increase as a function of mean TL [39] due to the relatively faster metabolic loss of ^14^N compared to ^15^N leaving animals at higher trophic levels with higher δ^15^N values [17]. Carbon isotopes (δ^13^C) in marine environments can be traced from basal resources such as particulate organic matter (POM) including phytoplankton, to consumers to determine primary carbon sources [37, 41]. These values are often used to distinguish between two geographically distinct food webs. Altabet and Francois [42] demonstrated that surface water δ^13^C values of POM lay at approximately -22 ‰ in temperate latitudes but decrease to -25 ‰, sometimes down to -35 ‰ [43] closer to Antarctica. Thus, animals feeding in Antarctic food webs demonstrate correspondingly low carbon isotope values [44–47], compared to those feeding in temperate food webs [48, 49]. The interpretation of bulk stable isotope (BSI) signals is, however, not without uncertainty. In addition to the prey type and foraging range, the trophic position (TP) is known to be influenced by endogenous factors such as nutritional stress, metabolic activity of tissues, diet quality, body size, excretory mechanisms and feeding rate [26–30]. Further, the isotopic signals associated with tissues of different biomolecular composition (i.e. lipids, carbohydrates, proteins) have frequently been recorded [30–32]. The extent to which tissue types within an individual differ in their δ^15^N and δ^13^C values carries inherent uncertainty for robust quantification of diet and represents a methodological aspect of cetacean dietary investigation that has not been thoroughly addressed.

In cetacean research, blubber and skin tissue are the most commonly used tissue types for dietary investigation as they are metabolically active and can easily be obtained via non-lethal biopsies from healthy, free-swimming individuals [50, 51]. Marine mammal blubber is principally composed of lipids and contains small amounts of protein [30, 52]. By contrast, skin mainly contains protein and limited amounts of lipids [30, 53, 54]. In BSIA, lipids confound analyses by decreasing the tissue ^13^C/^12^C and hence lowering measured δ^13^C values [31]. As lipids are depleted in ^13^C relative to proteins and carbohydrates [31], tissues are often treated to account for and minimise the potential impact on δ^13^C that can interfere with BSIA interpretation. Two approaches are commonly used to account for lipids. The first methods is the physical removal of lipid fractions through solvent extraction prior to BSIA. Alternatively, where the relationship between lipid-containing and lipid-depleted tissues of a species is known, mathematical corrections have been developed and applied [4, 55, 56].

In an effort to further strengthen data obtained from long-term monitoring of SHHW diet, the current study sought to compare the BSI measurements obtained from lipid-adjusted blubber and skin tissues respectively. In order to test the hypothesis that δ^13^C and δ^15^N values of blubber and skin taken from the same individual could be used interchangeably, 171 paired samples were investigated, providing new insights into method application, data interpretations, and species physiology.

## 2. Material and methods

### 2.1 Sample collection

Blubber and skin biopsy samples were obtained for long-term monitoring under the HWSP from free-swimming SHHW of the east coast of Australia-migrating stock (E1 as defined by the International Whaling Commission; [35], between 2008 and 2018. The biopsies were collected off North Stradbroke Island, southeast Queensland, Australia (approximately 27°26 S, 153°34 E) during the annual northward (June/ July) and southward (September/ October) E1 HW migration. Biopsy samples were collected using a modified 0.22 calibre rifle Paxarms NZ, Domett, New Zealand) with flotation darts. Darts were fired as recommended by Lambertsen et al. [57], from the research vessel and aimed at the ventral and slightly posterior area to the dorsal fin, then stored onboard on ice until sub-sectioned and transferred to a -18 freezer for long-term storage. For more details, see Bengtson Nash et al. [58].

The collection of samples was carried out under a Scientific Purposes permit, granted by the QLD department of Environment and Heritage Protection and an animal ethics permit granted by the Griffith University Animal Ethics Committee. In total, 171 paired blubber and skin biopsy samples were included in this study. Blubber tissue was lipid extracted with solvents prior to analysis while skin tissue was mathematically lipid corrected. Both are referred to as “lipid-adjusted” in subsequent text.

### 2.2 Lipid adjustment

#### 2.2.1 Solvent extraction

Approximately 30 mg of blubber was lipid extracted prior to BSIA. The solvent lipid extraction of blubber tissue was completed using a modified methanol-dichloromethane-water (2:1:0.8 v/v/v MeOH/CH_2_Cl_2_/H_2_O) method pioneered by Bligh and Dyer [59], as described in detail elsewhere (e.g. Groβ et al. [4]).

#### 2.2.2 Mathematical correction

Previously, Groβ et al. [4] determined the most appropriate isotopic discrimination factor of skin for the study population to be 8.92 ‰. The mass balance approach developed by Fry [60], was considered the best fit for lipid correction of SHHW skin, and was therefore applied in this study. The correction applied in this study was as follows (Eq 1):

$$\delta^{13}C_{LFM}=\delta^{13}C_{B}+D\;x\;\left(1-\frac{C:N_{LF}}{C:N_{B}}\right)$$ (1)

Where δ^13^C_LFM_ is the lipid-corrected carbon isotope value of skin, δ^13^C_B_ is the bulk carbon isotope value measured from SHHW skin, and D is the isotopic discrimination factor. C: N_LF_ is the measured ratio of lipid-corrected skin tissue, whilst C: N_B_ is the measured ratio of bulk SHHW skin tissue.

### 2.3 Bulk stable isotope analysis

Skin tissue and lipid-extracted blubber tissue were oven dried overnight at approximately 58°C and pulverized in to 1–2 mg samples which were placed into tin capsules for δ^13^C and δ^15^N analysis. Stable isotope abundances were calculated in permil using the following (Eq 2):

δX=[(Rsample/Rstandard)–1]x1000 (2)

Where, X is ^13^C or ^15^N, and R is the respective ratio ^13^C/^12^C or ^15^N/^14^N. The international reference standards used for carbon and nitrogen are, respectively, Vienna Pee Dee Belemnite and N_2_ in air. Laboratory standards, sucrose and (NH_4_)_2_SO_4_ were calibrated using international standards IAEA-CH_6_ for carbon and IAEA N1 for nitrogen. The preparation system used is a Europa EA-GSL interfaced to a SERCON Hydra 20–20 isotope ratio mass-spectrometer (IRMS). Based on analysis of replicate standards, the standard deviations for δ^13^C and δ^15^N averaged 0.1 ‰ and 0.15 ‰, respectively.

### 2.4 Krill range calculation

The krill range i.e., the isotopic range expected for individual whale δ^13^C and δ^15^N values feeding exclusively on Antarctic krill, was calculated based on δ^13^C and δ^15^N isotopic values including (+/- SD) of krill derived from Eisenmann et al. [33]. Blubber and skin trophic fractionation (TF) estimates were calculated in this study applying values from S1 and S2 Tables to S2 Equation in S1 File. The krill range for lipid-extracted blubber was -28.14 to -24.66 and 5.96 to 9.34, while for lipid-corrected skin the range was -27.09 to -23.61 and 5.12 to 8.50 respectively for δ^13^C and δ^15^N. This facilitated comparison of blubber and skin foraging results, allowing for an inter-annual evaluation of diet representation within and between tissues throughout sample years.

### 2.5 Trophic position calculation

The estimated trophic position for SHHWs lipid-extracted blubber and lipid-corrected skin tissue relative to krill (S1 Table in S1 File) was calculated applying the trophic level calculation as described in S1 Equation in S1 File. As tissue-specific TF values for SHHWs was not available, the authors applied the TF value derived from fin whale (*Balaenoptera physalus*) skin tissue (2.82%) from Borrell et al. [61]. This provided an opportunity to investigate whether the isotopic signature relationship between blubber and skin would be reflected in their estimated TP values.

### 2.6 Statistics

Data analyses were performed in R version 1.3. 1093 [62] and GraphPad Prism version 9.0.2 [63]. A Shapiro-Wilk test and a Levene’s test were used to test the data for normality and homogeneity of variance, respectively. All statistical results were interpreted using a significance level of α = 0.05. The δ^13^C and δ^15^N isotopic values across sex and migration showed no significant difference (p = 0.1841 and p = 0.1184 respectively), thus all samples were treated as a homogenous cohort. A Shapiro-wilks test demonstrated non-normality for δ^13^C and δ^15^N isotopic values within and between lipid-adjusted blubber and skin, thus non-parametric statistical tests were further applied. Two separate Wilcoxon matched pair signed rank tests were used to test for differences in δ^13^C and δ^15^N values between the two tissue types. The test structure used δ^13^C and δ^15^N as test variables for differences in the factor ‘tissue type’ with fixed values for lipid-adjusted blubber and skin tissue. A non-parametric Kruskal-Wallis test with multiple comparisons was applied to investigate trends across sample years for δ^13^C and δ^15^N values.

## 3. Results and discussion

The present study is the first to investigate tissue specific BSI measurements and implications for interpretation of SHHW diet. Our results showed that there are significant differences in δ^13^C and δ^15^N values obtained from lipid-adjusted blubber and skin from the same individuals. Such differences were more prominent in some individuals, thus occasionally led to different down-stream interpretation of trophic position. There was greater variability in δ^15^N values of lipid-extracted blubber compared to lipid-corrected skin. The tissue-specific variation in δ^15^N values was similarly reflected in tissue-specific TP estimates as lipid-adjusted blubber and skin tissue which demonstrated a TP of 3.65, and 3.29, respectively. These findings underscore that tissue-specific variation must be thoroughly investigated before comparing dietary results obtained via BSIA using two different tissues and caution against interchangeable use of tissues or comparison between them.

### 3.1 Bulk differences

**Fig 1:**
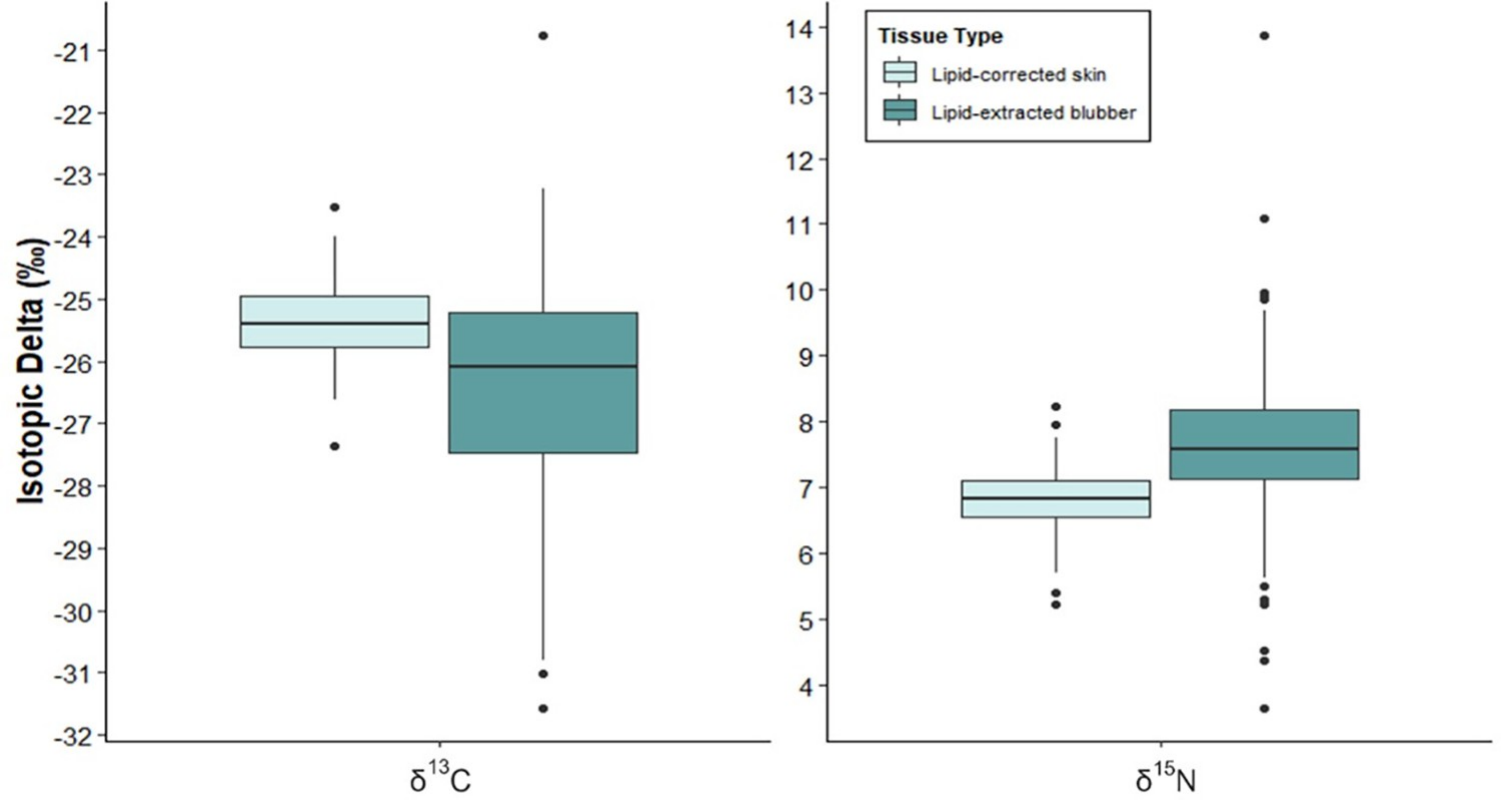
Box plot showing the distribution of δ^13^C and δ^15^N values for lipid-extracted blubber and lipid-corrected skin tissue (n = 171).

For both δ^13^C and δ^15^N values of lipid-adjusted tissues, significant differences were observed (δ^13^C p = 0.0001 and δ^15^N p = 0.0001; Fig 1). Lipid-extracted blubber values showed greater variability for both δ^13^C and δ^15^N compared to lipid-corrected skin (Table 1., Fig 1).

**Table 1:** Table overview of the mean, standard deviation (SD) and range for δ^13^C and δ^15^N values for lipid-extracted blubber and lipid-corrected skin tissue of E1 humpback whales (n = 171).

Lipid-corrected skin					Lipid-extracted blubber				
Isotope	n	Mean	SD	Range	Isotope	n	Mean	SD	Range
C	171	-25.35	0.60	-27.35 - -23.52	C	171	-26.40	1.83	-31.56 - -20.77
N	171	6.81	0.46	5.21–8.22	N	171	7.65	1.14	3.65–13.87

As the tissues were obtained from the same individual whale, the extent of the variability in both isotope signatures was not expected. There is limited research on the comparison of δ^13^C and δ^15^N values between HW blubber and skin tissue, however, a significant difference between the two tissues either for one or both isotopes has been documented (e.g. Todd et al. [54], and Groβ et al. [4]). However, the reasons for this variation are not clear, and thus we attempt to evaluate several factors that may have contributed to the significant differences found in this study.

### 3.2 Inter-annual differences

**Fig 2:**
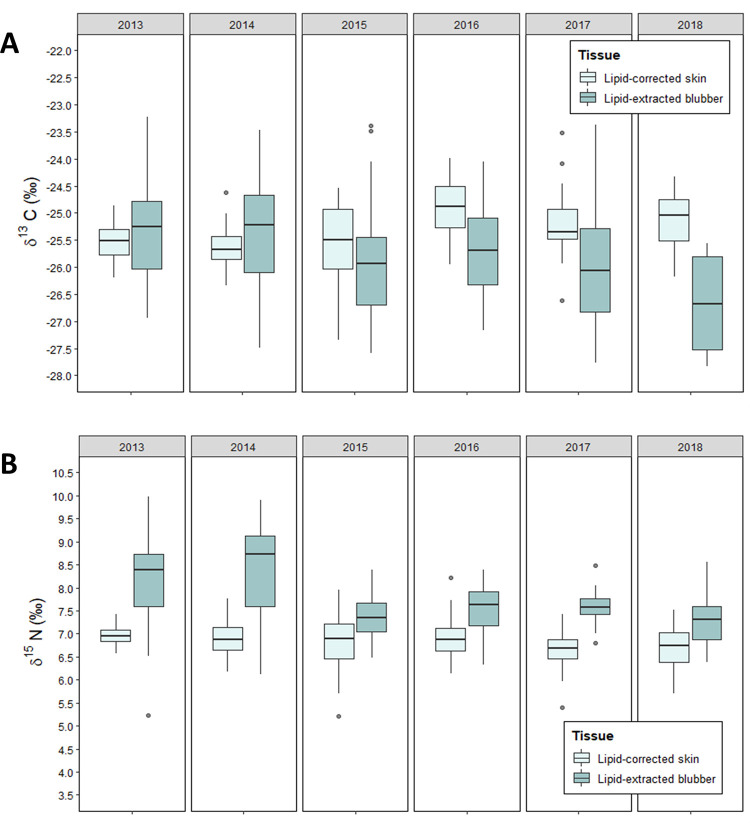
Isotopic values of blubber and skin (n = 171) across all sample years. (A) illustrates comparison between both tissues for δ^13^C and (B) for δ^15^N values.

Large inter-annual variability in isotopic signatures has previously been evidenced via fatty acid analysis for this population [64]. When samples were separated by year, limiting analysis to those years where >10 paired samples were available for analysis (2013–2018), significant inter-annual differences were observed in selected years. For the six years in which comparisons were possible, three years demonstrated a significant difference in the δ^13^C values between the two tissue types (2016; p = 0.0216, 2017; p = 0.0335 and 2018; p = 0.0001; Fig 2A). Similarly, three years, albeit three different years, showed significant differences in δ^15^N values between tissue types (2013; p = 0.0003, 2014; p = 0.0001 and 2017; p = 0.0001; Fig 2B).

As the significant differences in δ^13^C and δ^15^N values between lipid-adjusted blubber and skin did not occur in the same sample years, there may be underlaying tissue-specific variations that could be driving the variability in isotope signatures. Fig 2 illustrates an overall low variability in both δ^13^C and δ^15^N values for lipid-corrected skin across all sample years, compared to lipid-extracted blubber that has a greater variability with more prominent oscillations in some years like 2014. The differences in isotopic signatures between the tissues may lead to issues for interpretation because we cannot be certain whether the variability present in blubber δ^13^C and δ^15^N values is caused by variability in prey type or foraging location, or whether the observed variability is introduced by endogenous factors or method artefacts. Hence, we do not know if we lose information about foraging variability when we just interpret results from skin, or if we introduce variability to results when we just interpret results from blubber tissue.

### 3.3 Trophic position comparison

**Fig 3:**
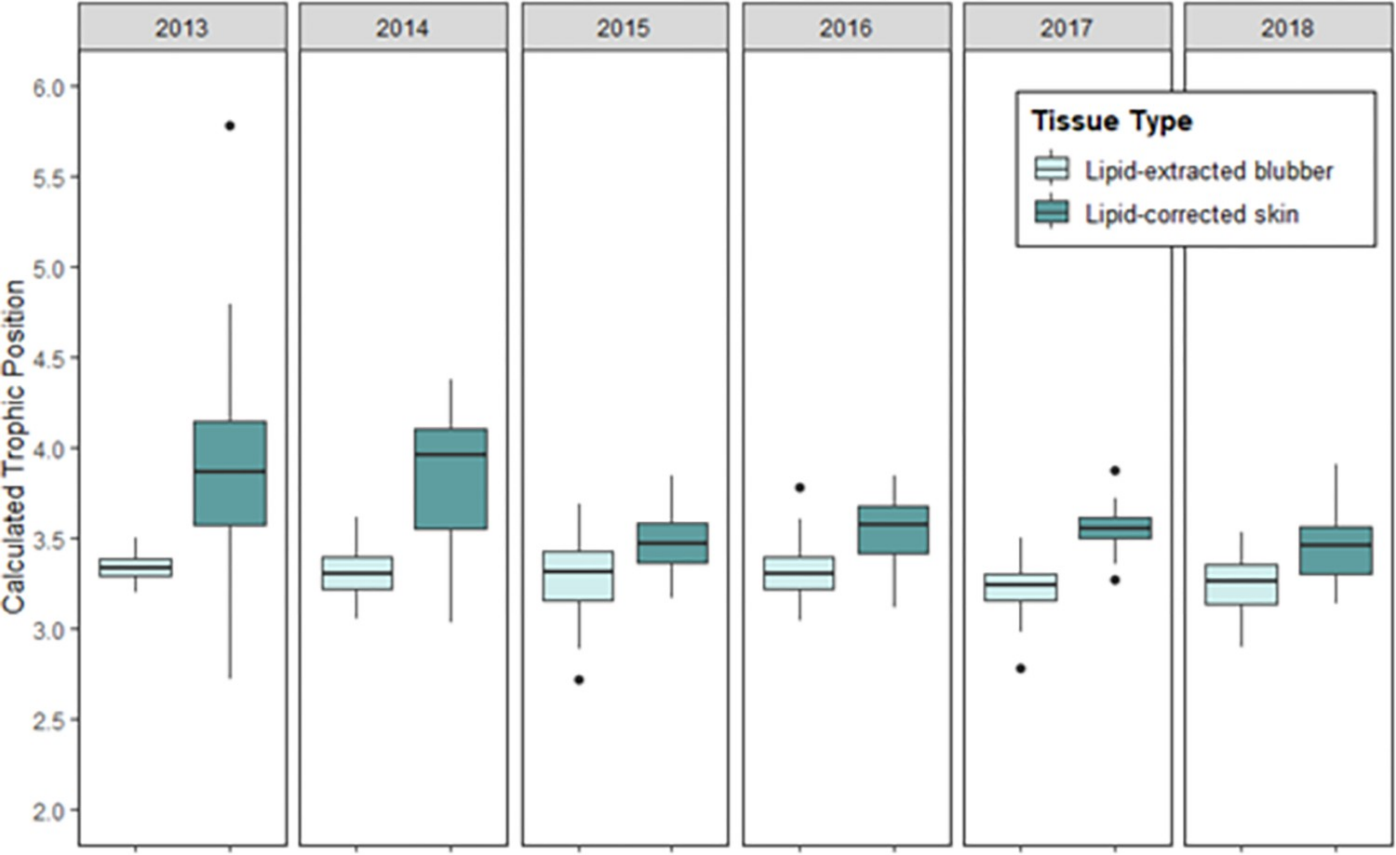
Trophic position estimates for blubber and skin tissue across sample years; 2013–2018.

Trophic position estimates were calculated to investigate whether the observed differences between blubber and skin δ^13^C and δ^15^N values also leads to differences in the dietary information derived from the two tissue types. For continuation with focus on samples with years where >10 paired samples were available for analysis (2013–2018), a strong significant difference was observed two tissues (Welch t-test p = 2.2_e_-16). This variation was observed for all said sample years (2013; p = 0.00083, 2014; p = 3.995e-08, 2015; p = 3.529e-05, 2016; p = 6.785e-05, 2017; p = 5.74e-12, and 2018; p = 0.001149; Fig 3).

The observed overall significant difference in δ^15^N values between lipid-adjusted blubber and skin tissue, was reflected in the TP values calculated for both tissues (blubber = 3.65±0.47, and skin = 3.29 ±0.16). The calculated TP values are congruent with the classical feeding paradigm of a high-fidelity krill diet in SHHWs [65–68]. However, as some variation was observed in 2013, 2014 and 2016 (Fig 3), there is reason to question whether the variation in δ^15^N values between the tissues can be linked to variation in TP interpretation.

The equation used to estimate TP has limitations, which can lead to errors in interpretation. Firstly, the discussion of diet composition and trophic position depends on an accurate estimate of stable isotope enrichment of δ^15^N between humpback whales and their prey. Unfortunately, there are presently no published trophic enrichment factors for humpback whales. The trophic fractionation factor (Δ^15^N) used in the equation, 2.82 ‰ for lipid-extracted blubber and lipid corrected-skin tissue, are only based on estimates. Additionally, Δ^15^N vary between and within species and tissues, introducing error when estimates are based on other tissues or species. Secondly, an average δ^15^N value for krill was used in the equation, which introduces errors as there are spatial and temporal differences in δ^15^N values of krill [69–72]. Some introduced uncertainty could be reduced by analysing compound specific nitrogen isotope composition of amino acids [70–72], however we were unable to analyse compound specific isotopes due to cost restrains.

### 3.4 Tissue-specific krill space

**Fig 4:**
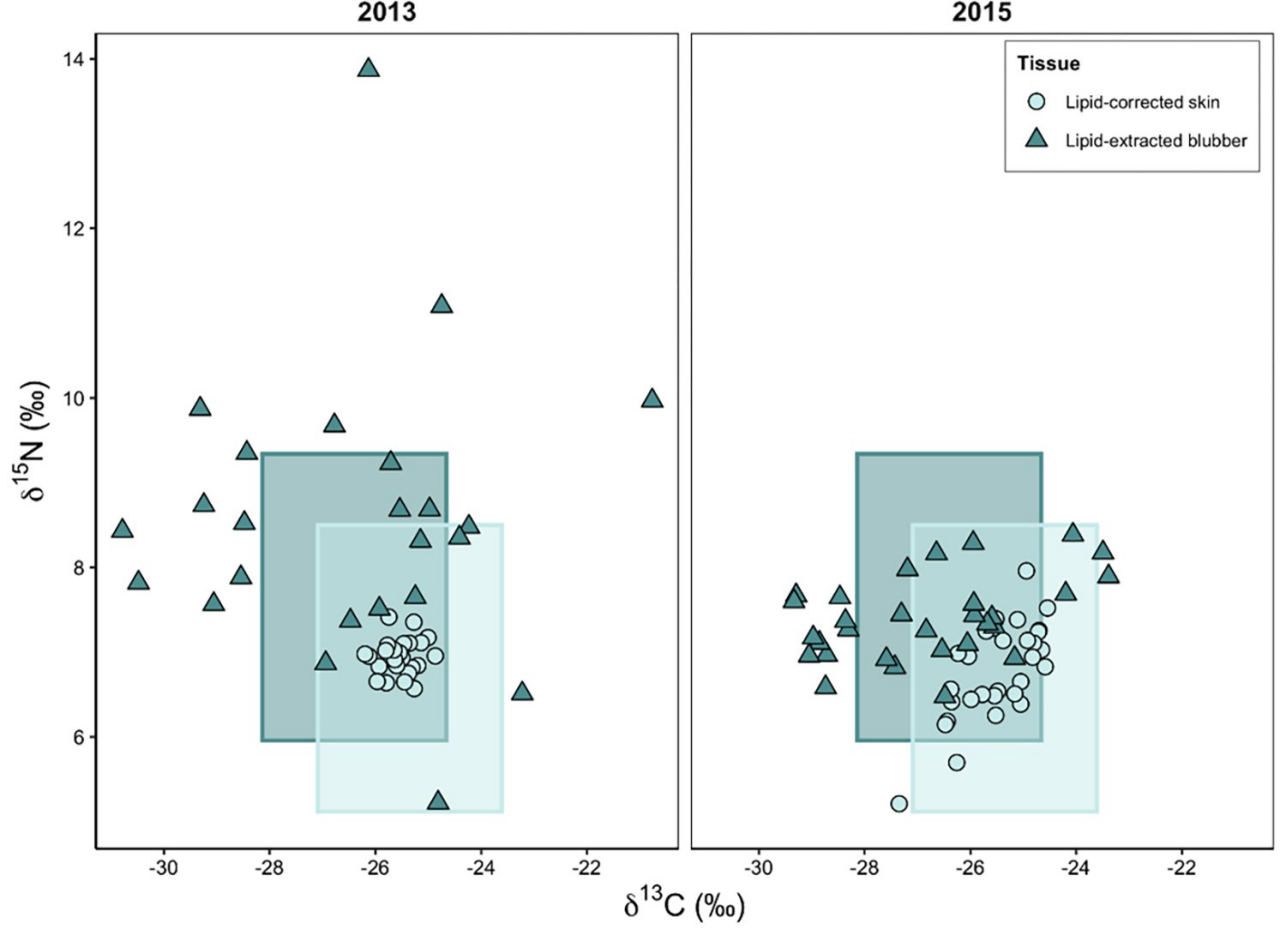
Scatterplot illustrating the tissue-specific krill space for δ^13^C and δ^15^N values of lipid-adjusted blubber and skin tissue of 2013 (n = 24) and 2015 (n = 30).

The implications of tissue-specific variability in BSI values for the interpretation of diet was further investigated by creating a krill space (isotope range) for each tissue. The shaded areas in Fig 4 illustrate the tissue-specific krill space in which SHHW δ^13^C and δ^15^N values are expected to fall if the individual whales were feeding primarily on krill the austral summer prior to sampling. The figure only shows the δ^13^C and δ^15^N values of lipid-adjusted blubber and skin from two sample years, 2013 and 2015, as these years highlight the two different scenarios that we have observed between 2008 and 2020; lipid-corrected skin isotope values fall within the calculated krill space while either the majority of both δ^13^C and δ^15^N values of lipid-extracted blubber fall outside the krill space or the majority of just δ^13^C values of blubber fall outside the krill space.

For lipid-extracted blubber, in 2013 only 29.2% and 53.3% of the isotopic data points fell within the krill space in 2013 and 2015, respectively. This was half of what was observed for lipid-corrected skin, where 100% of the data fell within the krill space in 2013, and 96.7% in 2015. This leads to different interpretations about the diet plasticity of SHHW. If we would make inferences based on skin isotope results, we would conclude that SHHW exclusively feed on krill in the Southern Ocean. However, if we would only interpret blubber isotope results, we would conclude that SHHW exhibit a much greater diet plasticity than expected for a high-fidelity krill diet species. Interestingly, the observed variability does translate into different interpretations of TP.

### 3.5 Factors influencing variability

#### 3.5.1 Endogenous factors

To properly interpret stable isotope signatures of animal tissues, it is essential to account for temporal dynamics of isotopic integration such as tissue turn-over rate and diet-tissue incorporation.

Isotopic turnover time describes the time it takes for the isotopic value of the diet/ prey to be reflected in a tissue [73, 74]. Isotopic turnover time can vary within or among individuals, where overall body size, and both growth of new tissue and amount of tissue replacement due to metabolic turnover play fundamental roles in determining isotopic turnover rates [32, 75]. The turnover time for blubber and skin of SHHW is unknown, however SHHW blubber turnover is suggested to be <9 months, because the blubber lipid store is almost entirely depleted over the course of their annual migration, due to prolonged fasting [68]. Isotopic turnover time for skin δ^15^N has been estimated to be approximately 180 days for bottlenose dolphins (*Tursiops truncates*) and 163 days for blue whales (*Balaenoptera musculus*), while δ^13^C has been estimated to be approximately 104 days for bottlenose dolphins [76, 77]. Based on taxonomy and size, we therefore expect SHHW skin to have an isotopic turnover time that ranges from approximately 104 to 180 days. In the present study, there were roughly 60 days between E1 humpback whales leaving their Antarctic feeding grounds in March and the time they were sampled in June/ July, and roughly 150 days until they were sampled in September/ October. Although, turnover time for either SHHW tissue is unknown, we can assume that both tissues reflect a similar diet intake timeframe based on available information.

Aside from tissue turnover differences, the variation in δ^13^C and δ^15^N values between lipid-adjusted skin and blubber tissues may be linked to differences in tissue-specific metabolic routing, which is expected to produce a consistent offset between the stable isotope values of individual tissues. Metabolic routing of different biomolecules during tissue synthesis and metabolism impacts diet-tissue isotope discrimination. This means that some tissues may primarily reflect individual diet components such as carbohydrates and lipids derived from one dietary source and proteins derived from another [78, 79]. By way of example, a study by Misra [80] on bottlenose dolphins found that blubber tissue likely represents metabolic patterns linked to fatty acids and ketogenic amino acids related to fat synthesis and deposition within the tissue, whilst skin showed metabolites involved in gluconeogenic pathways pointing to active anabolic energy-generating metabolism. By extension, it is possible that the δ^13^C and δ^15^N values of SHHW blubber tissue may be drawn from a more direct energy pool, where lipids are immediately stored in the blubber, while secondary pathways may be involved in the growth of skin tissue. The complexity of tissue-specific metabolic routing and discrimination can also lead to uncertainty in lipid normalization models due to unknown protein-lipid discrimination values.

#### 3.5.2 Artefacts

In addition to endogenous factors, methodological artefacts should also be considered as a source of variation. The observed differences between both tissues may be related to the lipid-adjustment approaches applied to the respective tissue type. The mass-balance mathematical lipid correction model proposed by Fry [60] relies on precision, accuracy and reliability in predicting the lipid-free δ^13^C values. The model is based on C:N ratios and thus lipid content, which was estimated to have a mean standard error of ~0.05 in predicting lipid-free δ^13^C values for skin tissue of E1 humpback whales [4]. A study by Groß et al. [4] specifically calculated the discrimination value ‘D’ for skin tissue of individual E1 humpback whales to be applied in the mass balance correction model, which gave a ‘D’ value of 8.92 ‰ and a C:N_LM_ of 3.1. The authors recommended the use of these values in conjunction with the mass balance model for E1 humpback whale skin tissue, if the skin tissue has a low lipid content, leading to small lipid corrections that limit errors in interpretation. However, the use of these exact coefficient values for ‘D’ and C:N_LM_ increases uncertainty if the correction is applied to species and populations where empirical values are unknown. Thus, although the ‘D’ value has been determined for E1 humpback whales, the accuracy of the value is unknown, given the large interannual variability in δ^13^C and δ^15^N values (e.g. Bengtson Nash et al. [1], Tieszen et al. [32], and McConnaughey and McRoy [81]).

As with mathematical lipid correction, solvent-extraction may be similarly susceptible to the introduction of methodological artefacts. The dichloromethane/methanol solvent combination used in this study has been reported to have little influence on δ^15^N values [82]. However, a study by Murry et al. [83] on fish muscle tissue demonstrated that both δ^13^C and δ^15^N values of lipid-extracted samples had a significant enrichment of the heavier isotope relative to the non-extracted samples. Other studies, utilizing various solvent combinations for lipid extraction have detected fluctuations in δ^15^N values as a result of solvents interfering with structural components of the tissue [55, 84–86]. This could be linked to observations made throughout this study, where lipid-extracted blubber showed notable higher values for δ^15^N isotopic values and overall greater variation compared to lipid-corrected skin. Although, as the specific C:N ratio of lipid-extracted blubber was not measured in this study, it is difficult to indicate whether or not, or the extent of the samples still containing lipid post-extraction (e.g. Tatsch et al. [87], and references therein). In addition, all figures indicate a high variability and range in lipid-extracted blubber tissue. An increase in δ^15^N resulting from solvent lipid extractions has been linked to the loss of nitrogenous components such as amino acids (AA), which may be extracted unintentionally from the tissue as the solvents can remove polar and non-polar compounds in the process [88]. The hypothesis is that methanol, which removes mostly polar structural fat components that are attached to proteins, also removes amino acids at the same time as structural fats, resulting in enrichment of ^15^N (Murry et al. [83], and references therein). Although this study did not seek to address this method component, altered δ^15^N values post extraction have previously been observed in fish tissues; muscle and whole body samples [89], and liver tissues [82]. A study by Ryan et al. [56] found significant increases in δ^15^N values post lipid extraction for blubber of fin whales and skin of minke whales (*Balaenoptera acutorostrata*), where the overall changes were more prominent in blubber than skin tissue, which is logical given the respective lipid proportions. Thus, we hypothesis that E1 humpback whale blubber, being an adipose tissue with high lipid content is susceptible to solvent extraction related removal of amino acids resulting in the possibility of distorting the signal of δ^15^N values in BSIA.

## 4. Conclusion

This study showed that the overall comparison of lipid adjusted blubber and skin δ^13^C and δ^15^N values of SHHW were similar, but not to the extent that we can confidently recommend the interchangeable use of both tissues in this field of research. Although the mean trophic position of each year cohort was similar, the greater variability observed in blubber, which may be interpreted as higher trophic level feeding, is not present in skin values. This variability has been related to variation in lipid content, solvent interference, isotopic discrimination, and metabolic pathways between blubber and skin tissue of which due to limited resources and funds were not further explored or investigated in detail in this study. All are key factors that can impact the interpretation of stable isotope results. We recommend that future studies incorporate a standard for SHHW blubber and skin tissue, with the application of multiple lipid standardization approaches. Additionally, we suggest the inclusion of multiple solvent lipid extraction trials for blubber tissue to determine the potential impact on isotopic signatures. This will allow for optimization of dietary investigation and standardization of methodologies, which will improve long-term monitoring of SHHWs to provide new insights into energy utilisation by these populations.

## Supporting information

S1 File (DOCX)

S1 Data (PDF)

## References

[1] Bengtson Nash S. M. et al., “Signals from the south; humpback whales carry messages of Antarctic sea-ice ecosystem variability,” *Glob*. *Chang*. *Biol*., vol. 24, no. 4, pp. 1500–1510, Apr. 2017, doi: 10.1111/gcb.14035 29284198

[2] Druskat A., Ghosh R., Castrillon J., and Bengtson Nash S. M., “Sex ratios of migrating southern hemisphere humpback whales: A new sentinel parameter of ecosystem health,” 2019, doi: 10.1016/j.marenvres.2019.104749 31256980

[3] Waugh C. A, Nichols P. D, Noad M. C, and Nash S. B, “Lipid and fatty acid profiles of migrating Southern Hemisphere humpback whales *Megaptera novaeangliae*,” *Mar*. *Ecol*. *Prog*. *Ser*., vol. 471, pp. 271–281, 2012, doi: 10.3354/meps10059

[4] Groß J., Fry B., Burford M. A., and Bengtson Nash S., “Assessing the effects of lipid extraction and lipid correction on stable isotope values (δ13C and δ15N) of blubber and skin from southern hemisphere humpback whales,” *Rapid Commun*. *Mass Spectrom*., vol. 35, no. 16, pp. 1–11, 2021, doi: 10.1002/rcm.9140 34097783

[5] Castrillon J. and Bengtson Nash S., “Evaluating cetacean body condition; a review of traditional approaches and new developments,” *Ecology and Evolution*, vol. 10, no. 12. John Wiley and Sons Ltd, pp. 6144–6162, Jun. 01, 2020, doi: 10.1002/ece3.6301 32607220 PMC7319165

[6] Nicol S., “Krill, Currents, and Sea Ice: Euphausia superba and Its Changing Environment,” Oxford Academic, Feb. 2006. doi: 10.1641/0006-3568(2006)056[0111:KCASIE]2.0.CO;2

[7] Hofmann E. E. and Murphy E. J., “Advection, krill, and Antarctic marine ecosystems,” *Antarct*. *Sci*., vol. 16, no. 4, pp. 487–499, 2004, doi: 10.1017/S0954102004002275

[8] Stammerjohn S., Massom R., Rind D., and Martinson D., “Regions of rapid sea ice change: An inter-hemispheric seasonal comparison,” *Geophys*. *Res*. *Lett*., vol. 39, no. 6, pp. 1–8, 2012, doi: 10.1029/2012GL050874

[9] Kawaguchi S. et al., “Risk maps for Antarctic krill under projected Southern Ocean acidification,” *Nat*. *Clim*. *Chang*., vol. 3, no. 9, pp. 843–847, 2013, doi: 10.1038/NCLIMATE1937

[10] Meredith M. P. and King J. C., “Rapid climate change in the ocean west of the Antarctic Peninsula during the second half of the 20th century,” *Geophys*. *Res*. *Lett*., vol. 32, no. 19, pp. 1–5, 2005, doi: 10.1029/2005GL024042

[11] Teschke M., Wendt S., Kawaguchi S., Kramer A., and Meyer B., “A circadian clock in antarctic krill: An endogenous timing system governs metabolic output rhythms in the euphausid species Euphausia superba,” *PLoS One*, vol. 6, no. 10, 2011, doi: 10.1371/journal.pone.0026090 22022521 PMC3189233

[12] Meyer B. and Teschke M., Physiology of Euphausia superba, Volume 1. Kiel, Germany: Springer, 2016.

[13] Tulloch V. J. D., Plagányi É. E., Brown C., Richardson A. J., and Matear R., “Future recovery of baleen whales is imperiled by climate change,” *Glob*. *Chang*. *Biol*., vol. 25, no. 4, pp. 1263–1281, Apr. 2019, doi: 10.1111/gcb.14573 30807685 PMC6850638

[14] Seyboth E., Groch K. R., Dalla Rosa L., Reid K., Flores P. A. C., and Secchi E. R., “Southern Right Whale (Eubalaena australis) Reproductive Success is Influenced by Krill (Euphausia superba) Density and Climate,” Sci. Rep., vol. 6, pp. 1–9, 2016, doi: 10.1038/srep28205 27306583 PMC4910057

[15] Reilly S. et al., “Biomass and energy transfer to baleen whales in the South Atlantic sector of the Southern Ocean,” *Deep Sea Res*. *Part II Top*. *Stud*. *Oceanogr*., vol. 51, no. 12–13, pp. 1397–1409, 2004.

[16] Ware C. et al., “Bottom side-roll feeding by humpback whales (Megaptera novaeangliae) in the southern Gulf of Maine, U.S.A,” *Mar*. *Mammal Sci*., vol. 30, no. 2, pp. 494–511, 2014, doi: 10.1111/mms.12053

[17] Fry B., *Stable isotope ecology*. New York: Springer Science + Buisness Media, LLC, 2008.

[18] Watt C. Aand Ferguson S. H., “Fatty acids and stable isotopes (δ 13 C and δ 15 N) reveal temporal changes in narwhal (*Monodon monoceros*) diet linked to migration patterns,” *Mar. Mammal Sci*., vol. 31, no. 1, pp. 21–44, Jan. 2015, doi: 10.1111/mms.12131

[19] Bridge E. S. et al., “Bird migration and avian influenza: A comparison of hydrogen stable isotopes and satellite tracking methods,” *Ecol*. *Indic*., vol. 45, pp. 266–273, Oct. 2014, doi: 10.1016/j.ecolind.2014.04.027 25045322 PMC4097340

[20] Robillard A., Gauthier G., Therrien J.-F., Fitzgerald G., Provencher J. F., and Bêty J., “Variability in stable isotopes of snowy owl feathers and contribution of marine resources to their winter diet,” *J*. *Avian Biol*., vol. 48, no. 6, pp. 759–769, Jun. 2017, doi: 10.1111/jav.01257

[21] Kaczensky P. et al., “Stable isotopes reveal diet shift from pre-extinction to reintroduced Przewalski’s horses,” *Nature*, vol. 7, no. 5950, 2017, doi: 10.1038/s41598-017-05329-6 28729625 PMC5519547

[22] Chiaradia A., Ramírez F., Forero M. G., and Hobson K. A., “Stable Isotopes (δ13C, δ15N) Combined with Conventional Dietary Approaches Reveal Plasticity in Central-Place Foraging Behavior of Little Penguins Eudyptula minor,” *Front. Ecol. Evol*., vol. 3, no. JAN, p. 154, Jan. 2016, doi: 10.3389/fevo.2015.00154

[23] Borrell A., Gómez-Campos E., and Aguilar A., “Influence of Reproduction on Stable-Isotope Ratios: Nitrogen and Carbon Isotope Discrimination between Mothers, Fetuses, and Milk in the Fin Whale, a Capital Breeder,” *Physiol*. *Biochem*. *Zool*., vol. 89, no. 1, pp. 41–50, Jan. 2016, doi: 10.1086/684632 27082523

[24] Pethybridge H. et al., “A global meta-analysis of marine predator nitrogen stable isotopes: Relationships between trophic structure and environmental conditions,” *Glob*. *Ecol*. *Biogeogr*., vol. 27, no. 9, pp. 1043–1055, Sep. 2018, doi: 10.1111/geb.12763

[25] Connolly R. M. and Waltham N. J., “Spatial analysis of carbon isotopes reveals seagrass contribution to fishery food web,” *Ecosphere*, vol. 6, no. 9, p. art148, Sep. 2015, doi: 10.1890/ES14-00243.1

[26] Hobson K. A. and Welch H. E., “Cannibalism and trophic structure in a high Arctic lake: insights from stable-isotope analysis,” *Can*. *J*. *Fish*. *Aquat*. *Sci*., vol. 52, no. 6, pp. 1195–1201, 1995, doi: 10.1139/f95-116

[27] Ponsard S. and Averbuch P., “Should growing and adult animals fed on the same diet show different δ^15^N values?,” *Rapid Commun*. *Mass Spectrom*., vol. 13, no. 13, pp. 1305–1310, 1999, doi: 10.1002/(SICI)1097-0231(19990715)13:13&lt;1305::AID-RCM654&gt;3.0.CO;2-D. 10407315

[28] Overman N. C. and Parrish D. L., “Stable isotope composition of walleye: ^15^N accumulation with age and area-specific differences in δ^13^C,” *Can*. *J*. *Fish*. *Aquat*. *Sci*., vol. 58, no. 6, pp. 1253–1260, 2001, doi: 10.1139/f01-072

[29] Vanderklift M. A. and Ponsard S., “Sources of variation in consumer-diet δ^15^N enrichment: A meta-analysis,” *Oecologia*, vol. 136, no. 2, pp. 169–182, 2003, doi: 10.1007/s00442-003-1270-z 12802678

[30] Cherry S. G., Derocher A. E., Hobson K. A., Stirling I., and Thiemann G. W., “Quantifying dietary pathways of proteins and lipids to tissues of a marine predator,” *J*. *Appl*. *Ecol*., vol. 48, no. 2, pp. 373–381, 2011, doi: 10.1111/j.1365-2664.2010.01908.x

[31] DeNiro M. J. and Epstein S., “Influence of diet on the distribution of carbon isotopes in animals,” *Microw*. *Opt*. *Technol*. *Lett*., vol. 42, pp. 495–506, 1978, doi: 10.1002/mop.25285

[32] Tieszen L. L., Boutton T. W., Tesdahl K. G., and Slade N. A., “Fractionation and turnover of stable carbon isotopes in animal tissues: Implications for δ^13^C analysis of diet,” *Oecologia*, vol. 57, no. 1–2, pp. 32–37, 1983, doi: 10.1007/BF00379558 28310153

[33] Eisenmann P., Fry B., Holyoake C., Coughran D., Nicol S., and Bengtson Nash S., “Isotopic evidence of a wide spectrum of feeding strategies in Southern hemisphere humpback whale baleen records,” *PLoS One*, vol. 11, no. 5, pp. 1–20, 2016, doi: 10.1371/journal.pone.0156698 27244081 PMC4887117

[34] Schwarz D., Spitzer S. M., Thomas A. C., Kohnert C. M., Keates T. R., and Acevedo-Gutiérrez A., “Large-scale molecular diet analysis in a generalist marine mammal reveals male preference for prey of conservation concern,” *Ecol*. *Evol*., vol. 8, no. 19, pp. 9889–9905, Oct. 2018, doi: 10.1002/ece3.4474 30386584 PMC6202700

[35] Acevedo J. et al., “Evidence of spatial structuring of eastern South Pacific humpback whale feeding grounds,” *Endanger*. *Species Res*., vol. 22, no. 1, pp. 33–38, 2013, doi: 10.3354/esr00536

[36] Witteveen B. H., Worthy G. A. J., Foy R. J., and Wynne K. M., “Modeling the diet of humpback whales: An approach using stable carbon and nitrogen isotopes in a Bayesian mixing model,” *Mar*. *Mammal Sci*., vol. 28, no. 3, pp. 233–250, 2012, doi: 10.1111/j.1748-7692.2011.00508.x

[37] Zuev A. G. et al., “Stable Isotope Trophic Fractionation (13C/12C and 15N/14N) in Mycophagous Diptera Larvae,” *Biol*. *Bull*., vol. 46, no. 5, pp. 457–465, 2019, doi: 10.1134/S1062359019050157

[38] Mill A. C., Pinnegar J. K., and Polunin N. V. C., “Explaining isotope trophic-step fractionation: Why herbivorous fish are different,” *Funct*. *Ecol*., vol. 21, no. 6, pp. 1137–1145, 2007, doi: 10.1111/j.1365-2435.2007.01330.x

[39] Minagawa M. and Wada E., “Stepwise enrichment of ^15^N along food chains: Further evidence and the relation between δ^15^N and animal age,” *Geochim*. *Cosmochim*. *Acta*, vol. 48, no. 5, pp. 1135–1140, 1984, doi: 10.1016/0016-7037(84)90204-7

[40] Trites A. W., *Marine mammal trophic levels and trophic interactions*, 3rd ed., no. September 2018. Elsevier Ltd., 2019.

[41] Seyboth E., Botta S., Mendes C. R. B., Negrete J., Dalla Rosa L., and Secchi E. R., “Isotopic evidence of the effect of warming on the northern Antarctic Peninsula ecosystem,” *Deep*. *Res*. *Part II Top*. *Stud*. *Oceanogr*., vol. 149, no. December 2017, pp. 218–228, 2018, doi: 10.1016/j.dsr2.2017.12.020

[42] Altabet M. A. and Francois R., “Sedimentary nitrogen isotopic ratio as a recorder for surface ocean nitrate utilization,” *Global Biogeochem*. *Cycles*, vol. 8, no. 1, pp. 103–116, 1994, doi: 10.1029/93GB03396

[43] Goericke R. and Fry B., “Variations of marine plankton in δ^13^N with latitude, temperature, and dissolved CO_2_ in the world ocean,” *Glob*. *Biochem*. *Cycles*, vol. 8, no. 1, pp. 85–90, 1994.

[44] Wada E., Terazaki M., Kabaya Y., and Nemoto T., “^15^N and ^13^C abundances in the Antartic Ocean with emphasis on the biogeochemical structure of the food web,” *Deep Sea Res*. Part A, Oceanogr. Res. Pap., vol. 34, no. 5–6, pp. 829–841, 1987, doi: 10.1016/0198-0149(87)90039-2

[45] Cherel Y., “Isotopic niches of emperor and Adélie penguins in Adélie Land, Antarctica,” pp. 813–821, 2008, doi: 10.1007/s00227-008-0974-3

[46] Hall-Aspland S. A., Hall A. P., and Rogers T. L., “A new approach to the solution of the linear mixing model for a single isotope: Application to the case of an opportunistic predator,” *Oecologia*, vol. 143, no. 1, pp. 143–147, 2005, doi: 10.1007/s00442-004-1783-0 15599768

[47] Hodum P. J. and Hobson K. A., “Trophic relationships among Antarctic fulmarine petrels: insights into dietary overlap and chick provisioning strategies inferred from stable-isotope (δ15N and δ13C) analysis,” *Mar*. *Ecol*. *Prog*. *Ser*., vol. 198, pp. 273–281, 2000.

[48] Davenport S. Rand Bax N. J, “A trophic study of a marine ecosystem off southeastern Australia using stable isotopes of carbon and nitrogen,” *Can*. *J*. *Fish*. *Aquat*. *Sci*., vol. 59, no. 3, pp. 514–530, 2002, doi: 10.1139/f02-031

[49] Harris B. P., Young J. W., Revill A. T., and Taylor M. D., “Understanding diel-vertical feeding migrations in zooplankton using bulk carbon and nitrogen stable isotopes,” *J*. *Plankton Res*., vol. 36, no. 4, pp. 1159–1163, 2014, doi: 10.1093/plankt/fbu026

[50] Budge S. M., Iverson S. J., and Koopman H. N., “Studying trophic ecology in marine ecosystems using fatty acids: A primer on analysis and interpretation,” *Mar*. *Mammal Sci*., vol. 22, no. 4, pp. 759–801, 2006, doi: 10.1111/j.1748-7692.2006.00079.x

[51] Noren D. P. and Mocklin J. A., “Review of cetacean biopsy techniques: Factors contributing to successful sample collection and physiological and behavioral impacts,” *Mar*. *Mammal Sci*., vol. 28, no. 1, pp. 154–199, 2012, doi: 10.1111/j.1748-7692.2011.00469.x

[52] Castrillon J., Huston W., and Bengtson Nash S., “The blubber adipocyte index: A nondestructive biomarker of adiposity in humpback whales (Megaptera novaeangliae),” *Ecol*. *Evol*., vol. 7, no. 14, pp. 5131–5139, 2017, doi: 10.1002/ece3.2913 28770053 PMC5528216

[53] Filatova O. A. et al., “The diets of humpback whales (Megaptera novaeangliae) on the shelf and oceanic feeding grounds in the western North Pacific inferred from stable isotope analysis,” *Mar*. *Mammal Sci*., vol. 29, no. 3, pp. 253–265, 2013, doi: 10.1111/j.1748-7692.2012.00617.x

[54] Todd S., Ostrom P., Lien J., and Abrajano J., “Use of biopsy samples of humpback whale (Megaptera novaeangliae) skin for stable isotope (δ13C) determination,” *J*. *Northwest Atl*. *Fish*. *Sci*., vol. 22, no. December 1997, pp. 71–76, 1997, doi: 10.2960/J.v22.a6

[55] Post D. M., Layman C. A., Arrington D. A., Takimoto G., Quattrochi J., and Montaña C. G., “Getting to the fat of the matter: Models, methods and assumptions for dealing with lipids in stable isotope analyses,” *Oecologia*, vol. 152, no. 1, pp. 179–189, 2007, doi: 10.1007/s00442-006-0630-x 17225157

[56] Ryan C., McHugh B., Trueman C. N., Harrod C., Berrow S. D., and O’Connor I., “Accounting for the effects of lipids in stable isotope (δ13C and δ15N values) analysis of skin and blubber of balaenopterid whales,” *Rapid Commun*. *Mass Spectrom*., vol. 26, no. 23, pp. 2745–2754, 2012, doi: 10.1002/rcm.6394 23124665

[57] Lambertsen R. H., Baker C. S., Weinrich M., and Modi W. S., “An improved whale biopsy system designed for multidisciplinary research,” CRC Press, no. In Nondestructive biomarkers in vertebrates, pp. 219–244, 2020.

[58] Bengtson Nash S. M., “Toxicological risks and considerations associated with lipophilic contaminant burdens of Southern Ocean mysticetes,” *Mar*. *mammal Ecotoxicol*., pp. 381–400, 2018.

[59] Bligh E. G. and Dyer W. J., “A rapid method of total lipid extraction and purification,” *Can*. *J*. *Biochem*. *Physiol*., vol. 37, no. 8, 1959. doi: 10.1139/o59-099 13671378

[60] Fry B., “Stable isotopic indicators of habitat use by Mississippi River Fish,” *J*. *North Am*. *Benthol*. *Soc*., vol. 21, no. 4, pp. 676–685, 2002.

[61] Borrell A., Abad-Oliva N., Gõmez-Campos E., Giménez J., and Aguilar A., “Discrimination of stable isotopes in fin whale tissues and application to diet assessment in cetaceans,” *Rapid Commun*. *Mass Spectrom*., vol. 26, no. 14, pp. 1596–1602, 2012, doi: 10.1002/rcm.6267 22693115

[62] R Core Team, “Integrated development for R.,” *RStudio*, vol. 42. RStudio PBC, Boston, MA, p. 14, 2020, [Online]. Available: https://rstudio.com.

[63] S. GraphPad, “GraphPad Prism.” GraphPad, San Diego, California, 2020, [Online]. Available: www.graphpad.com.

[64] Groß J., Virtue P., Nichols P. D., Eisenmann P., Waugh C. A., and Nash S. B., “Interannual variability in the lipid and fatty acid profiles of east Australia ‑ migrating humpback whales (Megaptera novaeangliae) across a 10 ‑ year timeline,” *Sci. Rep*., pp. 1–14, 2020, doi: 10.1038/s41598-020-75370-5 33106590 PMC7589506

[65] Witteveen B. H., Worthy G. A. J., Wynne K. M., Hirons A. C., Andrews A. G., and Markel R. W., “Trophic levels of North Pacific Humpback whales (*Megaptera novaeangliae*) through analysis of stable isotopes: Implications on prey and resource quality,” *Aquat. Mamm*., vol. 37, no. 2, pp. 101–110, 2011, doi: 10.1578/AM.37.2.2011.101

[66] Haro D., Sabat P., Arreguín-Sánchez F., Neira S., and Hernández-Padilla J. C., “Trophic role of the humpback whale (Megaptera novaeangliae) in the feeding area of Magellan Strait, Chile,” *Ecol*. *Indic*., vol. 109, p. 105796, 2020, doi: 10.1016/j.ecolind.2019.105796

[67] Paterson R. A., Paterson P., and Cato D. H., “Status of humpback whales, *Megaptera novaeangliae*, in east Australia at the end of the 20th century,” *Mem*. *MUSEUM*, vol. 2, no. 47, pp. 579–586, 2001.

[68] Chittleborough R. G., “Dynamics of two populations of the humpback whale. Megaptera novaeangliae (borowski),” *Mar*. *Freshw*. *Res*., vol. 16, no. 1, pp. 33–128, 1965, doi: 10.1071/MF9650033

[69] Polito M. J., Reiss C. S., Trivelpiece W. Z., Patterson W. P., and Emslie S. D., “Stable isotopes identify an ontogenetic niche expansion in Antarctic krill (Euphausia superba) from the South Shetland Islands, Antarctica,” *Mar*. *Biol*., vol. 160, no. 6, pp. 1311–1323, 2013, doi: 10.1007/s00227-013-2182-z

[70] Chikaraishi Y. et al., “Determination of aquatic food-web structure based on compound-specific nitrogen isotopic composition of amino acids,” *Limnol*. *Oceanogr*. *Methods*, vol. 7, no. NOV, pp. 740–750, 2009, doi: 10.4319/lom.2009.7.740

[71] Chikaraishi Y., Kashiyama Y., Ogawa N. O., Kitazato H., and Ohkouchi N., “Metabolic control of nitrogen isotope composition of amino acids in macroalgae and gastropods: Implications for aquatic food web studies,” *Mar*. *Ecol*. *Prog*. *Ser*., vol. 342, pp. 85–90, 2007, doi: 10.3354/meps342085

[72] McClelland J. W. and Montoya J. P., “Trophic relationships and the nitrogen isotopic composition of amino acids in plankton,” *Ecology*, vol. 83, no. 8, pp. 2173–2180, 2002, doi: 10.1890/0012-9658(2002)083[2173:TRATNI]2.0.CO;2.

[73] Zilversmith D, Entenman C, and Fishler C, “On the calculation of ‘turnover time’ and ‘turnover rate’ from experiments involving the use of labeling agents,” *Gen*. *Physiol*., pp. 325–331, 1942. 10.1085/jgp.26.3.325 PMC2142557 19873346

[74] Reiner J. M., “The study of metabolic turnover rates by means of isotopic tracers: I. Fundamental relations,” *Arch*. *Biochem*. *Biophys*., vol. 46, no. 1, pp. 53–79, 1953, 10.1016/0003-9861(53)90170-2. 13092947

[75] Newsome S. D., Clementz M. T., and Koch P. L., “Using stable isotope biogeochemistry to study marine mammal ecology,” *Mar*. *Mammal Sci*., vol. 26, no. 3, pp. 509–572, 2010, doi: 10.1111/j.1748-7692.2009.00354.x

[76] Browning N. E., Dold C., I-Fan J., and Worthy G. A. J., “Isotope turnover rates and diet-tissue discrimination in skin of ex situ bottlenose dolphins (Tursiops truncatus),” *J*. *Exp*. *Biol*., vol. 217, no. 2, pp. 214–221, 2014, doi: 10.1242/jeb.093963 24072795

[77] Busquets-Vass G. et al., “Estimating blue whale skin isotopic incorporation rates and baleen growth rates: Implications for assessing diet and movement patterns in mysticetes,” *PLoS One*, vol. 5, no. 12, 2017, doi: 10.1371/journal.pone.0177880 28562625 PMC5451050

[78] Wolf N., Newsome S. D., Peters J., and Fogel M. L., “Variability in the routing of dietary proteins and lipids to consumer tissues influences tissue-specific isotopic discrimination,” *Rapid Commun*. *Mass Spectrom*., vol. 29, no. 15, pp. 1448–1456, 2015, doi: 10.1002/rcm.7239 26147485

[79] Caut S., Angulo E., and Courchamp F., “Variation in discrimination factors (Δ15N and Δ13C): The effect of diet isotopic values and applications for diet reconstruction,” *J*. *Appl*. *Ecol*., vol. 46, no. 2, pp. 443–453, 2009, doi: 10.1111/j.1365-2664.2009.01620.x

[80] Misra B. B., Mariel R. H. I., Ivonne H. B. G., Emanuel H. N., Raúl D. G., and Cristina C. D. R., “1H NMR metabolomic analysis of skin and blubber of bottlenose dolphins reveal a functional metabolic dichotomy,” *Comp. Biochem. Physiol.—Part D Genomics Proteomics*, vol. 30, no. February, pp. 25–32, 2019, doi: 10.1016/j.cbd.2019.02.004 30771562

[81] McConnaughey T. and McRoy C. P., “Food-Web structure and the fractionation of Carbon isotopes in the bering sea,” *Mar*. *Biol*., vol. 53, no. 3, pp. 257–262, 1979, doi: 10.1007/BF00952434

[82] Logan J. M. and Lutcavage M. E., “A comparison of carbon and nitrogen stable isotope ratios of fish tissues following lipid extractions with non-polar and traditional chloroform/methanol solvent systems,” *Rapid Commun*. *Mass Spectrom*., vol. 22, pp. 1081–1086, 2008, doi: 10.1002/rcm.3471 18327856

[83] Murry B. A., Farrell J. M., Teece M. A., and Smyntek P. M., “Effect of lipid extraction on the interpretation of fish community trophic relationships determined by stable carbon and nitrogen isotopes,” *Can*. *J*. *Fish*. *Aquat*. *Sci*., vol. 63, no. 10, pp. 2167–2172, 2006, doi: 10.1139/F06-116

[84] Sotiropoulos M. A., Tonn W. M., and Wassenaar L. I., “Effects of lipid extraction on stable carbon and nitrogen isotope analyses of fish tissues: Potential consequences for food web studies,” *Ecol*. *Freshw*. *Fish*, vol. 13, no. 3, pp. 155–160, 2004, doi: 10.1111/j.1600-0633.2004.00056.x

[85] Yurkowski D. J., Hussey N. E., Semeniuk C., Ferguson S. H., and Fisk A. T., “Effects of lipid extraction and the utility of lipid normalization models on δ^13^C and δ^15^N values in Arctic marine mammal tissues,” *Polar Biol*., vol. 38, no. 2, pp. 131–143, 2015, doi: 10.1007/s00300-014-1571-1

[86] Sweeting C. J., Polunin N. V. C., and Jennings S., “Effects of chemical lipid extraction and arithmetic lipid correction on stable isotope ratios of fish tissues,” *Rapid Commun*. *Mass Spectrom*., vol. 20, no. 4, pp. 595–601, 2006, doi: 10.1002/rcm.2347 16429479

[87] Tatsch A. C. C., Secchi E. R., and Botta S., “Effects of acidification, lipid removal and mathematical normalization on carbon and nitrogen stable isotope compositions in beaked whale (Ziphiidae) bone,” *Rapid Commun*. *Mass Spectrom*., vol. 30, no. 3, pp. 460–466, 2016, doi: 10.1002/rcm.7457 26754138

[88] Bearhop S., Waldron S., and Furness R. W., “Influence of Lipid and Uric Acid on δ^13^C and δ^15^N Values of Avian Blood: Implications for Trophic Studies,” *Auk*, vol. 117, no. 2, pp. 504–507, 2000, doi: 10.2307/4089734

[89] Logan J. M., Jardine T. D., Miller T. J., Bunn S. E., Cunjak R. A., and Lutcavage M. E., “Lipid corrections in carbon and nitrogen stable isotope analyses: Comparison of chemical extraction and modelling methods,” *J*. *Anim*. *Ecol*., vol. 77, no. 4, pp. 838–846, 2008, doi: 10.1111/j.1365-2656.2008.01394.x 18489570

