# Supplementary material for: Interpretation of southern hemisphere humpback whale diet via stable isotopes; implications of tissue-specific analysis
Source: PLoS One. 2023 Apr 3;18(4):e0283330. doi: 10.1371/journal.pone.0283330 (PMC10069779; doi:10.1371/journal.pone.0283330)
Supplement: S1 File — (DOCX) [file pone.0283330.s001.docx]

**Interpretation of southern hemisphere humpback whale diet via stable isotopes; implications of tissue -specific analysis**

June Eggebo*^a^, Jasmin Groβ ^a^ and Susan Bengtson Nash ^a^

^a^ Southern Ocean Persistent Organic Pollutants Program, Centre for Planetary Health and Food Security, School of Environment and Science, Griffith University, Nathan, QLD 4111, Australia

Corresponding author:

[s.bengtsonnash@griffith.edu.au](mailto:s.bengtsonnash@griffith.edu.au)

*Keywords:* Blubber, Foraging ecology, Lipids, Sentinel parameter, Skin, Trophic biology

**Overview:**

Number of Figures: 4 in manuscript.

Number of Tables: 1 in manuscript, 2 in SI (S1 and S2).

Number of Equations: 2 in manuscript, 2 in SI (S1 and S2).

Raw data information: See S3.0.

**S1.0 Trophic position calculation – Equations and data overview**

Equation S1) Tissue-specific trophic position calculation

Trophic position (TP) for SHHWs was calculated from lipid-adjusted blubber and skin tissues, relative to krill using the following equation:

TP = $2+\left( \delta^{15}N_{T}-\delta^{15}N_{A} \right)/\Delta^{15}N_{S}$

Where, 2 is the TP of the primary consumer, T is tissue type (lipid-extracted blubber or lipid-corrected skin), A is Antarctic krill (prey) and $\Delta^{15}N$ is the TF value. Mean lipid-corrected skin and lipid-extracted blubber δ^15^N isotope values were derived from BSIA in this study, the mean Antarctic krill δ^15^N value of 3.2 ‰ was derived from literature estimates as shown in Table S1, and the TF value was derived from fin whale (*Balaenoptera physalus*) skin tissue (2.82%) from Borrell et al [61].

**Table S1:** Antarctic krill estimates applied to trophic position calculation.

|  | **δ^13^C** | **SD** | **δ^15^N** | **SD** | **Literature** |
| --- | --- | --- | --- | --- | --- |
| Antarctic krill (*Euphausia superba*) | -27.10 | 1.74 | 3.20 | 1.69 | Eisenmann et al. [4] |

**S2.0 Krill space calculation – Equations and data overview**

*Equation S2) Tissue-specific krill space calculation*

${\delta X}_{\mathrm{AT}}={{\delta X}_{A}+TF}_{T}\pm\mathrm{SD}_{A}$

Where, δX is δ^13^C or δ^15^N, A is Antarctic krill (prey; Table S1.1), TF is trophic fractionation (Table S1.2), T is tissue type (blubber or skin), and SD is standard deviation. Based on this equation, minimum and maximum isotopic range values for either tissues was calculated (Table S2.1) and applied as krill-space estimates in figure 4 and 5.

**Table S2:** Tissue-specific krill space values.

| **Lipid-extracted blubber krill space** | | **Lipid-corrected skin krill space** | |
| --- | --- | --- | --- |
| δ^13^C | δ^15^N | δ^13^C | δ^15^N |
| -28.14 - -24.66 | 5.96 – 9.34 | -27.09 - -23.61 | 5.12 – 8.50 |

**S3.0 Raw data file access – Data Digital Object Identifiers (DOIs)**

The datasets generated and/or analysed during the current study are available in the Griffith Research Data repository, <https://doi.org/10.25904/1912/4431>. Contact for access rights: [s.bengtsonnash@griffith.edu.au](mailto:s.bengtsonnash@griffith.edu.au).
