# Supplementary material for: Interpretation of southern hemisphere humpback whale diet via stable isotopes; implications of tissue-specific analysis
Source: PLoS One. 2023 Apr 3;18(4):e0283330. doi: 10.1371/journal.pone.0283330 (PMC10069779; doi:10.1371/journal.pone.0283330)
Supplement: S1 Data — (PDF) [file pone.0283330.s002.pdf]

Completed by June Eggebo

Research Data in Brief

Article information

Article title
Southern hemisphere humpback whale lipid-adjusted blubber and skin biopsy data used for bulk stable isotope analysis to evaluate methodology utilized for long-term dietary monitoring.

Authors
June Eggebo*[a], Jasmin Groβ [a] and Susan Bengtson Nash [a]

Affiliations
[a] Southern Ocean Persistent Organic Pollutants Program, Centre for Planetary Health and Food Security, School of Environment and Science, Griffith University, Nathan, QLD 4111, Australia

Corresponding author's email address and Twitter handle
s.bengtsonnash@griffith.edu.au
twitter: @Antarctica_POPs

Keywords
Foraging ecology, Lipids, Sentinel parameter, Trophic biology, Stable Isotopes, Carbon, Nitrogen, Southern Ocean.

Abstract
Paired blubber and skin biopsy samples (n=171)were obtained for long-term monitoring under the humpback whale sentinel program (HWSP) from free-swimming southern hemisphere humpback whales (SHHWs) of the east coast of Australia-migrating stock between 2008 and 2018. The biopsies were collected off North Stradbroke Island, southeast Queensland, Australia (approximately 27°26 S, 153°34 E) during the annual northward (June/ July) and southward (September/ October) migration. Blubber tissue was lipid extracted with solvents prior to analysis while skin tissue was mathematically lipid corrected.

Both tissues were analysed for bulk stable isotopes; carbon ($\delta^{13}C$) and nitrogen ($\delta^{15}N$) analysis, which was measured in permil (‰). Isotopic values from paired blubber and skin samples from the same individuals were compared to assess whether tissues could be used interchangeably for isotope analysis and dietary interpretation. Significant differences were observed for both $\delta^{13}C$ and $\delta^{15}N$ values between lipid-extracted blubber and lipid-corrected skin. Were skin demonstrated little variation and more consistency in its distribution of data, whilst blubber showed great variability with some sample years indicating great oscillations for $\delta^{15}N$ values. Trophic position (TP) estimates were calculated to investigate whether the observed differences between blubber and skin $\delta^{13}C$ and $\delta^{15}N$ values also leads to differences in the dietary information derived from the two tissue types. Overall, when samples were considered as a single cohort, TP did not vary significantly between the two tissues. The implications of

tissue-specific variability in BSI values for the interpretation of diet was further investigated by creating a krill space (isotope range) for each tissue. The tissue-specific krill range would predict the range of which is expected for $\delta^{13}C$ and $\delta^{15}N$ values to lay within if the individual whale is feeding exclusively on prey item; Antarctic krill (*Euphausia superba*) as in accordance with the SHHW's classical feeding model. Two sample years, 2013 and 2015 was included in the visualisation as these years showed the greatest variability between the two tissues. Almost all lipid-corrected skin data points fell within the expected krill range of this tissue, whilst less than half of lipid-extracted blubber isotope data fell within the range expected this tissue.

The evaluation and comparison of $\delta^{13}C$ and $\delta^{15}N$ values in SHHW blubber and skin tissue contribute new methodologically based understanding of bulk stable isotope research. Not only does this research highlight the variation in $\delta^{13}C$ and $\delta^{15}N$ values found in lipid-extracted blubber and lipid-corrected skin tissue, but it further underscores the growing importance of emphasizing tissue selection criteria for improved accuracy of the interpretation of long-term dietary results of SHHWs and therefore monitoring of Southern Ocean health.

Specifications table

**Subject**	Animal Physiology
**Specific subject area**	North Stradbroke Island, southeast Queensland, Australia
**Type of data**	Table<br>Figure<br>Equation
**How the data were acquired**	The preparation system used is a Europa EA-GSL interfaced to a SERCON Hydra 20–20 isotope ratio mass-spectrometer (IRMS).
**Data format**	Raw<br>Analyzed
**Description of data collection**	In situ biopsy sampling from free swimming southern hemisphere humpback whales. A total of 171 paired blubber and skin biopsies were obtained with a modified 0.22 calibre rifle (Paxarms NZ) and flotation darts. Biopsies were immediately sub-sectioned, with the lipid fraction sub-sectioned at blubber core depth 3-4 cm. Blubber and skin samples were stored on ice in the field, and then transferred to – 20 °C freezers until bulk stable isotope analysis (BSIA).<br><br>NB. Migration direction (south or north) and sex (male or female) was recorded for all sampled whales, however statistically, there was no difference in $\delta^{13}C$ and $\delta^{15}N$

	values within/ between tissues from either migration direction nor sex, thus these varables were not included in further analysing in this study.<br><br>Approximately 30 mg of blubber was lipid extracted prior to BSIA. The solvent lipid extraction of blubber tissue was completed using a modified methanol-dichloromethane-water (2:1:0.8 v/v/v MeOH/CH_2Cl_2/H_2O) method.<br><br>The mass balance approach for lipid-correction developed by Fry (2002) have been evaluated and suggested to be the best for for lipid correction for SHHW skin tissue according to Groß et al. (2021). Thus this model was applied in our study with the application of isotopic discrimination factor of 8.92 ‰.
**Data source location**	• *North Stradbroke Island*<br>• *Southeast Queensland*<br>• *Australia*<br>• Approximately 27°26 S, 153°34 E)
**Data accessibility**	*With the article*<br><br>Repository name: Griffith University Research Data<br>Publisher URI: https://www.griffith.edu.au/<br>DOI: https://doi.org/10.25904/1912/4431<br><br>**Item Access Status**<br>Contact for access rights: s.bengtsonnash@griffith.edu.au<br><br>Raw data available upon request.

Value of the data

- The data is useful as it is a long-term data collection of paired blubber and skin tissue samples from 2008-2018 used for bulk stable isotope analysis.
- Research involving long-term monitoring of SHHWs dietary trends and the health of the Southern Ocean though bulk stable isotope analysis and fatty acid research.
- Critical comparison of tissue-specific isotopic signals is, however, lacking resulting in uncertainty surrounding the representativeness and therefore utility of different tissues for accurate determination of recent foraging. This study therefore advances future methodological aspects of cetacean dietary analysis.

Data description

Manuscript:

<u>Figures</u>

Figure 1. Bulk difference: Box plot showing the distribution of δ13C and δ15N values for lipid-extracted blubber and lipid-corrected skin tissue (n=171).
Figure 2. *Inter-annual differences:* Isotopic values of blubber and skin (n=171) across all sample years. (A) illustrates comparison between both tissues for δ13C and (B) for δ15N values.
Figure 3. *Trophic position comparison:* Trophic position estimates for blubber and skin tissue across all sampling years.
Figure 4. *Tissue-specific krill space:* Scatterplot illustrating the tissue-specific krill space for δ13C and δ15N values of lipid-adjusted blubber and skin tissue of 2013 (n=24) and 2015 (n=30).

<u>Tables:</u>

Table 1: Bulk difference: Table overview of the mean, standard deviation (SD) and range for δ13C and δ15N values for lipid-extracted blubber and lipid-corrected skin tissue of E1 humpback whales (n=171).

Supplementary information document:
Eq. S1.S) Tissue-specific trophic position calculation
Eq. S2.0) Tissue-specific krill space calculation
Table S1.1) Antarctic krill estimates applied to trophic position calculation
Table S1.2) Calculated TP values applied to trophic position calculation.
Table S2.1) Tissue-specific krill space values

Data descriptions:
Sample data: uploaded as Excel doc. Also available as DOI under 'Experimental design, materials and methods' section.

Raw data: Raw data obtained from Mass Spectrometry BSIA Analysis from Stable Isotope Laboratory Griffith University, Nathan, QLD 4111, Australia.

Experimental design, materials and methods

All data analyses were performed in R version 1.3. 1093 (R Core Team, 2020) and GraphPad Prism version 9.0.2 (GraphPad, 2020).

DOI was created with Griffith University Research Data: <u>https://doi.org/10.25904/1912/4431</u>

Ethics statements
The collection of samples was carried out under Scientific Purposes permit, granted by the QLD department of Environment and Heritage Protection and animal ethics permit granted by the Griffith University Animal Ethics Committee.

Acknowledgments
This work was funded by a Griffith University Honours Student Grant. June Eggebo thanks the Centre for Planetary Health and Food Security for her Honours Thesis Write-up Scholarship. The authors acknowledge the support from the Griffith University Stable Isotope Laboratory staff and the contributions of field volunteers who assisted with sample collection.

Declaration of interests
The authors declare that they have no known competing financial interests or personal relationships that could have appeared to influence the work reported in this paper.

References

Acevedo, J., Haro, D., Dalla Rosa, L., Aguayo-Lobo, A., Hucke-Gaete, R., Secchi, E., Plana, J., Pastene, L., 2013. Evidence of spatial structuring of eastern South Pacific humpback whale feeding grounds. Endanger. Species Res. 22, 33–38. https://doi.org/10.3354/esr00536

Altabet, M.A., Francois, R., 1994. Sedimentary nitrogen isotopic ratio as a recorder for surface ocean nitrate utilization. Global Biogeochem. Cycles 8, 103–116. https://doi.org/10.1029/93GB03396

Bearhop, S., Waldron, S., Furness, R.W., 2000. Influence of Lipid and Uric Acid on $\delta^{13}C$ and $\delta^{15}N$ Values of Avian Blood: Implications for Trophic Studies. Auk 117, 504–507. https://doi.org/10.2307/4089734

Bengtson Nash, S.M., Castrillon, J., Eisenmann, P., Fry, B., Shuker, J.D., Cropp, R.A., Dawson, A., Bignert, A., Bohlin-Nizzetto, P., Waugh, C.A., Polkinghorne, B.J., Dalle Luche, G., McLagan, D., 2017. Signals from the south; humpback whales carry messages of Antarctic sea-ice ecosystem variability. Glob. Chang. Biol. 24, 1500–1510. https://doi.org/10.1111/gcb.14035

Bligh, E.G., Dyer, W.J., 1959. A rapid method of total lipid extraction and purification. Can. J. Biochem. Physiol. 37.

Borrell, A., Gómez-Campos, E., Aguilar, A., 2016. Influence of Reproduction on Stable-Isotope Ratios: Nitrogen and Carbon Isotope Discrimination between Mothers, Fetuses, and Milk in the Fin Whale, a Capital Breeder. Physiol. Biochem. Zool. 89, 41–50. https://doi.org/10.1086/684632

Bridge, E.S., Kelly, J.F., Xiao, X., Takekawa, J.Y., Hill, N.J., Yamage, M., Haque, E.U., Islam, M.A., Mundkur, T., Yavuz, K.E., Leader, P., Leung, C.Y.H., Smith, B., Spragens, K.A., Vandegrift, K.J., Hosseini, P.R., Saif, S., Mohsanin, S., Mikolon, A., Islam, A., George, A., Sivananinthaperumal, B., Daszak, P., Newman, S.H., 2014. Bird migration and avian influenza: A comparison of hydrogen stable isotopes and satellite tracking methods. Ecol. Indic. 45, 266–273. https://doi.org/10.1016/j.ecolind.2014.04.027

Browning, N.E., Dold, C., I-Fan, J., Worthy, G.A.J., 2014. Isotope turnover rates and diet-tissue discrimination in skin of ex situ bottlenose dolphins (*Tursiops truncatus*). J. Exp. Biol. 217, 214–221. https://doi.org/10.1242/jeb.093963

Budge, S.M., Iverson, S.J., Koopman, H.N., 2006. Studying trophic ecology in marine ecosystems using fatty acids: A primer on analysis and interpretation. Mar. Mammal Sci. 22, 759–801. https://doi.org/10.1111/j.1748-7692.2006.00079.x

Busquets-Vass, G., Newsome, S.D., Calambokidis, J., Serra-Valente, G., Jacobsen, J.K., Aguíñiga-García, S., Gendron, D., 2017. Estimating blue whale skin isotopic incorporation rates and baleen growth rates: Implications for assessing diet and movement patterns in mysticetes. PLoS One 5. https://doi.org/10.1371/journal.pone.0177880

Castrillon, J., Bengtson Nash, S., 2020. Evaluating cetacean body condition; a review of traditional approaches and new developments. Ecol. Evol. https://doi.org/10.1002/ece3.6301

Castrillon, J., Huston, W., Bengtson Nash, S., 2017. The blubber adipocyte index: A nondestructive biomarker of adiposity in humpback whales (*Megaptera novaeangliae*). Ecol. Evol. 7, 5131–5139. https://doi.org/10.1002/ece3.2913

Caut, S., Angulo, E., Courchamp, F., 2009. Variation in discrimination factors ($\Delta^{15}N$ and $\Delta^{13}C$): The effect of diet isotopic values and applications for diet reconstruction. J. Appl. Ecol. 46, 443–453. https://doi.org/10.1111/j.1365-2664.2009.01620.x

Cherel, Y., 2008. Isotopic niches of emperor and Adélie penguins in Adélie Land , Antarctica 813–821. https://doi.org/10.1007/s00227-008-0974-3

Cherry, S.G., Derocher, A.E., Hobson, K.A., Stirling, I., Thiemann, G.W., 2011. Quantifying dietary pathways of proteins and lipids to tissues of a marine predator. J. Appl. Ecol. 48, 373–381. https://doi.org/10.1111/j.1365-2664.2010.01908.x

Chiaradia, A., Ramírez, F., Forero, M.G., Hobson, K.A., 2016. Stable Isotopes (δ13C, δ15N) Combined with Conventional Dietary Approaches Reveal Plasticity in Central-Place Foraging Behavior of Little Penguins Eudyptula minor. Front. Ecol. Evol. 3, 154. https://doi.org/10.3389/fevo.2015.00154

Chikaraishi, Y., Kashiyama, Y., Ogawa, N.O., Kitazato, H., Ohkouchi, N., 2007. Metabolic control of nitrogen isotope composition of amino acids in macroalgae and gastropods: Implications for aquatic food web studies. Mar. Ecol. Prog. Ser. 342, 85–90. https://doi.org/10.3354/meps342085

Chikaraishi, Y., Ogawa, N.O., Kashiyama, Y., Takano, Y., Suga, H., Tomitani, A., Miyashita, H., Kitazato, H., Ohkouchi, N., 2009. Determination of aquatic food-web structure based on compound-specific nitrogen isotopic composition of amino acids. Limnol. Oceanogr. Methods 7, 740–750. https://doi.org/10.4319/lom.2009.7.740

Chittleborough, R.G., 1965. Dynamics of two populations of the humpback whale. *Megaptera novaeangliae* (borowski). Mar. Freshw. Res. 16, 33–128. https://doi.org/10.1071/MF9650033

Connolly, R.M., Waltham, N.J., 2015. Spatial analysis of carbon isotopes reveals seagrass contribution to fishery food web. Ecosphere 6, art148. https://doi.org/10.1890/ES14-00243.1

Davenport, S.R., Bax, N.J., 2002. A trophic study of a marine ecosystem off southeastern Australia using stable isotopes of carbon and nitrogen. Can. J. Fish. Aquat. Sci. 59, 514–530. https://doi.org/10.1139/f02-031

DeNiro, M.J., Epstein, S., 1978. Influence of diet on the distribution of carbon isotopes in animals. Microw. Opt. Technol. Lett. 42, 495–506. https://doi.org/10.1002/mop.25285

Druskat, A., Ghosh, R., Castrillon, J., Bengtson Nash, S.M., 2019. Sex ratios of migrating southern hemisphere humpback whales: A new sentinel parameter of ecosystem health. https://doi.org/10.1016/j.marenvres.2019.104749

Eisenmann, P., Fry, B., Holyoake, C., Coughran, D., Nicol, S., Bengtson Nash, S., 2016. Isotopic evidence of a wide spectrum of feeding strategies in Southern hemisphere humpback whale baleen records. PLoS One 11, 1–20. https://doi.org/10.1371/journal.pone.0156698

Filatova, O.A., Witteveen, B.H., Goncharov, A.A., Tiunov, A. V., Goncharova, M.I., Burdin, A.M., Hoyt, E., 2013. The diets of humpback whales (*Megaptera novaeangliae*) on the shelf and oceanic feeding grounds in the western North Pacific inferred from stable isotope analysis. Mar. Mammal Sci. 29, 253–265. https://doi.org/10.1111/j.1748-7692.2012.00617.x

Folch, J., Lees, M., Sloane Stanley, G.H., 1957. A simple method for the isolation and purification of total lipides from animal tissues. J. Biol. Chem. 226, 497–509. https://doi.org/10.1016/s0021-9258(18)64849-5

Fry, B., 2008. Stable isotope ecology. Springer Science + Buisness Media, LLC, New York. https://doi.org/10.1016/B978-0-12-409548-9.10915-7

Fry, B., 2002. Stable isotopic indicators of habitat use by Mississippi River Fish. J. North Am. Benthol. Soc. 21, 676–685.

Goericke, R., Fry, B., 1994. Variations of marine plankton in δ^{13}N with latitude, temperature, and dissolved CO_2 in the world ocean. Glob. Biochem. Cycles 8, 85–90.

GraphPad, S., 2020. GraphPad Prism.

Groß, J., Fry, B., Burford, M.., Bengtson Nash, S., n.d. Accounting for lipid interference when evaluating diet via stable isotope values in skin and blubber of southern hemisphere humpback whales.

Groß, J., Fry, B., Burford, M.A., Bengtson Nash, S., 2021. Assessing the effects of lipid extraction and lipid correction on stable isotope values (δ^{13}C and δ^{15}N) of blubber and skin from southern hemisphere humpback whales. Rapid Commun. Mass Spectrom. 35, 1–11. https://doi.org/10.1002/rcm.9140

Hall-Aspland, S.A., Hall, A.P., Rogers, T.L., 2005. A new approach to the solution of the linear mixing model for a single isotope: Application to the case of an opportunistic predator. Oecologia 143, 143–147. https://doi.org/10.1007/s00442-004-1783-0

Haro, D., Sabat, P., Arreguín-Sánchez, F., Neira, S., Hernández-Padilla, J.., 2020. Trophic role of the humpback whale (*Megaptera novaeangliae*) in the feeding area of Magellan Strait, Chile. Ecol. Indic. 109, 105796.

Harris, B.P., Young, J.W., Revill, A.T., Taylor, M.D., 2014. Understanding diel-vertical feeding migrations in zooplankton using bulk carbon and nitrogen stable isotopes. J. Plankton Res. 36, 1159–1163. https://doi.org/10.1093/plankt/fbu026

Hobson, K.A., Welch, H.E., 1995. Cannibalism and trophic structure in a high Arctic lake: insights from stable-isotope analysis. Can. J. Fish. Aquat. Sci. 52, 1195–1201. https://doi.org/10.1139/f95-116

Hodum, P.J., Hobson, K.A., 2000. Trophic relationships among Antarctic fulmarine petrels : insights into dietary overlap and chick provisioning strategies inferred from stable-isotope (δ^{15}N and δ^{13}C) analysis. Mar. Ecol. Prog. Ser. 198, 273–281.

Hofmann, E.E., Murphy, E.J., 2004. Advection, krill, and Antarctic marine ecosystems. Antarct. Sci. 16, 487–499. https://doi.org/10.1017/S0954102004002275

Kaczensky, P., Šturm, M.B., Sablin, M. V, Voigt, C.C., Smith, S., Ganbaatar, O., Balint, B., Walzer, C., Spasskaya, N.N., 2017. Stable isotopes reveal diet shift from pre-extinction to reintroduced Przewalski's horses. Nature 7. https://doi.org/10.1038/s41598-017-05329-6

Kawaguchi, S., Ishida, A., King, R., Raymond, B., Waller, N., Constable, A., Nicol, S., Wakita, M., Ishimatsu, A., 2013. Risk maps for Antarctic krill under projected Southern Ocean acidification. Nat. Clim. Chang. 3, 843–847. https://doi.org/10.1038/NCLIMATE1937

Logan, J.M., Jardine, T.D., Miller, T.J., Bunn, S.E., Cunjak, R.A., Lutcavage, M.E., 2008. Lipid corrections in carbon and nitrogen stable isotope analyses: Comparison of chemical extraction and modelling methods. J. Anim. Ecol. 77, 838–846. https://doi.org/10.1111/j.1365-2656.2008.01394.x

Logan, J.M., Lutcavage, M.E., 2008. A comparison of carbon and nitrogen stable isotope ratios of fish tissues following lipid extractions with non-polar and traditional chloroform/methanol solvent systems. Rapid Commun. Mass Spectrom. 22, 1081–1086. https://doi.org/10.1002/rcm

McClelland, J.W., Montoya, J.P., 2002. Trophic relationships and the nitrogen isotopic composition of amino acids in plankton. Ecology 83, 2173–2180. https://doi.org/10.1890/0012-9658(2002)083[2173:TRATNI]2.0.CO;2

McConnaughey, T., McRoy, C.P., 1979. Food-Web structure and the fractionation of Carbon isotopes in the bering sea. Mar. Biol. 53, 257–262. https://doi.org/10.1007/BF00952434

Meredith, M.P., King, J.C., 2005. Rapid climate change in the ocean west of the Antarctic Peninsula during the second half of the 20th century. Geophys. Res. Lett. 32, 1–5. https://doi.org/10.1029/2005GL024042

Meyer, B., Teschke, M., 2016. Physiology of Euphausia superba, Volume 1. ed. Springer, Kiel, Germany.

Mill, A.C., Pinnegar, J.K., Polunin, N.V.C., 2007. Explaining isotope trophic-step fractionation: Why herbivorous fish are different. Funct. Ecol. 21, 1137–1145. https://doi.org/10.1111/j.1365-2435.2007.01330.x

Minagawa, M., Wada, E., 1984. Stepwise enrichment of ^{15}N along food chains: Further evidence and the relation between δ^{15}N and animal age. Geochim. Cosmochim. Acta 48, 1135–1140. https://doi.org/10.1016/0016-7037(84)90204-7

Misra, B.B., Mariel, R.H.I., Ivonne, H.B.G., Emanuel, H.N., Raúl, D.G., Cristina, C.D.R., 2019. 1H NMR metabolomic analysis of skin and blubber of bottlenose dolphins reveal a functional metabolic dichotomy. Comp. Biochem. Physiol. - Part D Genomics Proteomics 30, 25–32. https://doi.org/10.1016/j.cbd.2019.02.004

Newsome, S.D., Clementz, M.T., Koch, P.L., 2010. Using stable isotope biogeochemistry to study marine mammal ecology. Mar. Mammal Sci. 26, 509–572. https://doi.org/10.1111/j.1748-7692.2009.00354.x

Nicol, S., 2006. Krill, Currents, and Sea Ice: Euphausia superba and Its Changing Environment, BioScience. Oxford Academic. https://doi.org/10.1641/0006-3568(2006)056[0111:KCASIE]2.0.CO;2

Noren, D.P., Mocklin, J.A., 2012. Review of cetacean biopsy techniques: Factors contributing to successful sample collection and physiological and behavioral impacts. Mar. Mammal Sci. 28, 154–199. https://doi.org/10.1111/j.1748-7692.2011.00469.x

Overman, N.C., Parrish, D.L., 2001. Stable isotope composition of walleye: ^{15}N accumulation with age and area-specific differences in δ^{13}C. Can. J. Fish. Aquat. Sci. 58, 1253–1260. https://doi.org/10.1139/f01-072

Paterson, R.A., Paterson, P., Cato, D.H., 2001. Status of humpback whales, *Megaptera novaeangliae*, in east Australia at the end of the 20th century. Mem. MUSEUM 2, 579–586.

Pethybridge, H., Choy, C.A., Logan, J.M., Allain, V., Lorrain, A., Bodin, N., Somes, C.J., Young, J., Ménard, F., Langlais, C., Duffy, L., Hobday, A.J., Kuhnert, P., Fry, B., Menkes, C., Olson, R.J., 2018. A global meta-analysis of marine predator nitrogen stable isotopes: Relationships between trophic structure and environmental conditions. Glob. Ecol. Biogeogr. 27, 1043–1055. https://doi.org/10.1111/geb.12763

Pethybridge, H.R., Parrish, C.C., Bruce, B.D., Young, J.W., Nichols, P.D., 2014. Lipid, fatty acid and energy density profiles of white sharks: Insights into the feeding ecology and ecophysiology of a complex top predator. PLoS One 9. https://doi.org/10.1371/journal.pone.0097877

Polito, M.J., Reiss, C.S., Trivelpiece, W.Z., Patterson, W.P., Emslie, S.D., 2013. Stable isotopes identify an ontogenetic niche expansion in Antarctic krill (*Euphausia superba*) from the South Shetland Islands, Antarctica. Mar. Biol. 160, 1311–1323. https://doi.org/10.1007/s00227-013-2182-z

Ponsard, S., Averbuch, P., 1999. Should growing and adult animals fed on the same diet show different δ^{15}N values? Rapid Commun. Mass Spectrom. 13, 1305–1310. https://doi.org/10.1002/(SICI)1097-0231(19990715)13:13<1305::AID-RCM654>3.0.CO;2-D

Post, D.M., Layman, C.A., Arrington, D.A., Takimoto, G., Quattrochi, J., Montaña, C.G., 2007. Getting to the fat of the matter: Models, methods and assumptions for dealing with lipids in stable isotope analyses. Oecologia 152, 179–189. https://doi.org/10.1007/s00442-006-0630-x

R Core Team, 2020. Integrated development for R. RStudio.

Reilly, S., Hedley, S., Borberg, J., Hewitt, R., Thiele, D., Watkins, J., Naganobu, M., 2004. Biomass and energy transfer to baleen whales in the South Atlantic sector of the Southern Ocean. Deep Sea Res. Part II Top. Stud. Oceanogr. 51, 1397–1409.

Reiner, J.M., 1953. The study of metabolic turnover rates by means of isotopic tracers: I. Fundamental relations. Arch. Biochem. Biophys. 46, 53–79. https://doi.org/https://doi.org/10.1016/0003-9861(53)90170-2

Robillard, A., Gauthier, G., Therrien, J.-F., Fitzgerald, G., Provencher, J.F., Bêty, J., 2017. Variability in stable isotopes of snowy owl feathers and contribution of marine resources to their winter diet. J. Avian Biol. 48, 759–769. https://doi.org/10.1111/jav.01257

Rolff, C., Elmgren, R., 2000. Use of riverine organic matter in plankton food webs of the Baltic Sea. Mar. Ecol. Prog. Ser. 197, 81–101. https://doi.org/10.3354/meps197081

Ryan, C., McHugh, B., Trueman, C.N., Harrod, C., Berrow, S.D., O'Connor, I., 2012. Accounting for the effects of lipids in stable isotope ($\delta^{13}C$ and $\delta^{15}N$ values) analysis of skin and blubber of balaenopterid whales. Rapid Commun. Mass Spectrom. 26, 2745–2754. https://doi.org/10.1002/rcm.6394

Schwarz, D., Spitzer, S.M., Thomas, A.C., Kohnert, C.M., Keates, T.R., Acevedo-Gutiérrez, A., 2018. Large-scale molecular diet analysis in a generalist marine mammal reveals male preference for prey of conservation concern. Ecol. Evol. 8, 9889–9905. https://doi.org/10.1002/ece3.4474

Seyboth, E., Botta, S., Mendes, C.R.B., Negrete, J., Dalla Rosa, L., Secchi, E.R., 2018. Isotopic evidence of the effect of warming on the northern Antarctic Peninsula ecosystem. Deep. Res. Part II Top. Stud. Oceanogr. 149, 218–228. https://doi.org/10.1016/j.dsr2.2017.12.020

Sotiropoulos, M.A., Tonn, W.M., Wassenaar, L.I., 2004. Effects of lipid extraction on stable carbon and nitrogen isotope analyses of fish tissues: Potential consequences for food web studies. Ecol. Freshw. Fish 13, 155–160. https://doi.org/10.1111/j.1600-0633.2004.00056.x

Stammerjohn, S., Massom, R., Rind, D., Martinson, D., 2012. Regions of rapid sea ice change: An inter-hemispheric seasonal comparison. Geophys. Res. Lett. 39, 1–8. https://doi.org/10.1029/2012GL050874

Teschke, M., Wendt, S., Kawaguchi, S., Kramer, A., Meyer, B., 2011. A circadian clock in antarctic krill: An endogenous timing system governs metabolic output rhythms in the euphausid species Euphausia superba. PLoS One 6. https://doi.org/10.1371/journal.pone.0026090

Tieszen, L.L., Boutton, T.W., Tesdahl, K.G., Slade, N.A., 1983. Fractionation and turnover of stable carbon isotopes in animal tissues: Implications for $\delta^{13}C$ analysis of diet. Oecologia 57, 32–37. https://doi.org/10.1007/BF00379558

Todd, S., Ostrom, P., Lien, J., Abrajano, J., 1997. Use of biopsy samples of humpback whale (*Megaptera novaeangliae*) skin for stable isotope ($\delta^{13}C$) determination. J. Northwest Atl. Fish. Sci. 22, 71–76. https://doi.org/10.2960/J.v22.a6

Trites, A.W., 2019. Marine mammal trophic levels and trophic interactions, 3rd ed, Encyclopedia of Ocean Sciences. Elsevier Ltd. https://doi.org/10.1016/B978-0-12-409548-9.11618-5

Vanderklift, M.A., Ponsard, S., 2003. Sources of variation in consumer-diet $\delta^{15}N$ enrichment: A meta-analysis. Oecologia 136, 169–182. https://doi.org/10.1007/s00442-003-1270-z

Wada, E., Terazaki, M., Kabaya, Y., Nemoto, T., 1987. ^{15}N and ^{13}C abundances in the Antartic Ocean with emphasis on the biogeochemical structure of the food web. Deep Sea Res. Part A, Oceanogr. Res. Pap. 34, 829–841. https://doi.org/10.1016/0198-0149(87)90039-2

Ware, C., Wiley, D.N., Friedlaender, A.S., Weinrich, M., Hazen, E.L., Bocconcelli, A., Parks, S.E., Stimpert, A.K., Thompson, M.A., Abernathy, K., 2014. Bottom side-roll feeding by humpback whales

(*Megaptera novaeangliae*) in the southern Gulf of Maine, U.S.A. Mar. Mammal Sci. 30, 494–511. https://doi.org/10.1111/mms.12053

Watt, C.A., Ferguson, S.H., 2015. Fatty acids and stable isotopes (δ ^{13} C and δ ^{15} N) reveal temporal changes in narwhal (*Monodon monoceros*) diet linked to migration patterns. Mar. Mammal Sci. 31, 21–44. https://doi.org/10.1111/mms.12131

Waugh, C.A., Nichols, P.D., Noad, M.C., Nash, S.B., 2012. Lipid and fatty acid profiles of migrating Southern Hemisphere humpback whales *Megaptera novaeangliae*. Mar. Ecol. Prog. Ser. 471, 271–281. https://doi.org/10.3354/meps10059

Witteveen, B.H., Worthy, G.A.J., Foy, R.J., Wynne, K.M., 2012. Modeling the diet of humpback whales: An approach using stable carbon and nitrogen isotopes in a Bayesian mixing model. Mar. Mammal Sci. 28, 233–250. https://doi.org/10.1111/j.1748-7692.2011.00508.x

Witteveen, B.H., Worthy, G.A.J., Wynne, K.M., Hirons, A.C., Andrews, A.G., Markel, R.W., 2011. Trophic levels of North Pacific Humpback whales (*Megaptera novaeangliae*) through analysis of stable isotopes: Implications on prey and resource quality. Aquat. Mamm. 37, 101–110. https://doi.org/10.1578/AM.37.2.2011.101

Wolf, N., Newsome, S.D., Peters, J., Fogel, M.L., 2015. Variability in the routing of dietary proteins and lipids to consumer tissues influences tissue-specific isotopic discrimination. Rapid Commun. Mass Spectrom. 29, 1448–1456. https://doi.org/10.1002/rcm.7239

Yurkowski, D.J., Hussey, N.E., Semeniuk, C., Ferguson, S.H., Fisk, A.T., 2015. Effects of lipid extraction and the utility of lipid normalization models on δ^{13}C and δ^{15}N values in Arctic marine mammal tissues. Polar Biol. 38, 131–143. https://doi.org/10.1007/s00300-014-1571-1

Zilversmith, D.., Entenman, C., Fishler, C., 1942. On the calculation of “turnover time” and “turnover rate” from experiments involving the use of labeling agents. Gen. Physiol. 325–331.

Zuev, A.G., Rozanova, O.L., Tsurikov, S.M., Panchenko, P.L., Ershova, M.A., Smolyarova, D.D., Krivosheina, M.G., Aleksandrova, A. V., Ivnitsky, S.B., Maleeva, Y. V., Tiunov, A. V., 2019. Stable Isotope Trophic Fractionation (^{13}C/^{12}C and ^{15}N/^{14}N) in Mycophagous Diptera Larvae. Biol. Bull. 46, 457–465. https://doi.org/10.1134/S1062359019050157
